# Influence of Cooking Methods on Flavor Parameters and Sensory Quality of Tibetan Sheep Meat Examined Using an Electronic Nose, an Electronic Tongue, GC–IMS, and GC–MS

**DOI:** 10.3390/foods14132181

**Published:** 2025-06-22

**Authors:** Shipeng Ge, Lijuan Han, Shengzhen Hou, Zhenzhen Yuan, Linsheng Gui, Shengnan Sun, Chao Yang, Zhiyou Wang, Baochun Yang

**Affiliations:** College of Agriculture and Animal Husbandry, Qinghai Univerity, Xining 810016, China; hlj880105@163.com (S.G.); 1987990009@qhu.edu.cn (S.H.); 2017990038@qhu.edu.cn (Z.Y.); 2017990039@qhu.edu.cn (L.G.); 2016980007@qhu.edu.cn (S.S.); yangchao@qhu.edu.cn (C.Y.); 1992990011@qhu.edu.cn (Z.W.); 1989990021@qhu.edu.cn (B.Y.)

**Keywords:** cooking method, Tibetan sheep, flavor, volatile flavor compounds

## Abstract

To explore the influence of cooking methods on the flavor parameters of Tibetan sheep, various techniques such as atmospheric-pressure (AP), high-pressure (HP), atmospheric-pressure high-pressure (APHP), and high-pressure atmospheric-pressure (HPAP) cooking were tested. The results indicated that APHP and HP cooking yielded the best sensory qualities, accounting for 26.15% and 25.51%, respectively. The HP group had the highest amino acid content at 34%, enhancing the meat’s sweet taste due to alanine, glycine, arginine, and methionine. Among 40 detected fatty acids, the order of saturated fatty acid (SFA), monounsaturated fatty acid (MUFA), polyunsaturated fatty acid (PUFA), and n-6/n-3 content was AP > APHP > HPAP > HP (*p* < 0.05). An electronic tongue and nose identified aroma components across the four cooking methods. Similarities in aroma were observed among the samples after cooking, while significant differences were found in the aroma components between the AP group and the other three cooking methods (*p* < 0.05). The gas chromatography–ion mobility spectrometry (GC–IMS) and gas chromatography–mass spectrometry (GC–MS) analyses of the meat in the four groups indicated that there were significant differences in volatile compounds among meat cooked with different methods (*p* < 0.05), with 56 and 365 flavor compounds detected by the two analytical techniques, respectively. Moreover, the GC–MS results indicated that the flavor substance content in the HP group accounted for 30.80% among these four sample groups. This comprehensive analysis showed that high-pressure steaming could significantly improve the flavor quality of Tibetan sheep, providing a theoretical basis and empirical reference for the optimization of pre-treatment conditions and the processing of Tibetan sheep.

## 1. Introduction

The Tibetan sheep (*Ovis aries*) is endemic to the Qinghai–Tibetan Plateau [1]. This species can be categorized into three distinct types—the high prototype, the valley type, and the Euler type—with the plateau type being the most prevalent variety. Qinghai Province is the principal region for Tibetan sheep production, accounting for 45% of China’s Tibetan sheep resources, and is consequently referred to as “the capital of Tibetan sheep in China”. The meat of the Tibetan sheep is distinguished by its lower fat content, high protein levels, milder flavor, and superior quality, freshness, tenderness, and taste compared to other lamb and sheep varieties [2].

Boiling is a prevalent method for cooking lamb in China and is highly favored by Chinese consumers. Flavor plays a crucial role in determining food quality and significantly influences consumer acceptance [3]. High-temperature heating not only modifies the texture of meat but also triggers flavor precursors to undergo a series of chemical reactions, resulting in distinctive aromas [4]. Simultaneously, volatile compounds are generated during the cooking process, exerting a significant influence on flavor, which in turn affects the overall quality and acceptability of the food [5]. These volatile compounds predominantly consist of acids, lipids, alcohols, aldehydes, and ketones, all of which possess low detection thresholds that are crucial for flavor development. This underscores the importance of comprehending and analyzing flavor compounds in food [6]. Furthermore, meat contains non-volatile substances produced during cooking that substantially contribute to the sensory attributes of sweetness, umami, and sourness [7]. These include specific amino acids and nucleotides that interact to create distinctive flavors in meat. However, there is a notable paucity of research on the factors influencing the taste and flavor of cooked Tibetan sheep.

Recent advancements in analytical techniques have enhanced the study of meat flavor, with electronic sensory analysis and gas chromatography–mass spectrometry (GC–MS) being the most frequently employed methods for flavor analysis. Electronic sensory analysis emulates the mammalian senses of taste and smell to detect food flavors, with the electronic nose system capable of identifying volatile substances within specific ranges with high sensitivity [8]. Gas chromatography–mass spectrometry (GC–MS) offers the advantages of requiring only small sample sizes, coupled with high sensitivity and rapid analysis [9], thereby facilitating detailed evaluations of specific components [10]. Zhang et al. employed a combination of GC–MS and electronic nose/tongue analysis to differentiate volatile compounds and taste characteristics between three plant-based beef analogs (PBBA) and beef, which led to the identification of the principal flavor-contributing substances [11].

Gas chromatography–ion mobility spectrometry (GC–IMS) represents an innovative technology for gas phase separation and detection, integrating the separation capabilities of gas chromatography (GC) with the rapid detection speed of ion mobility spectrometry (IMS) [12]. This has led to its extensive application in detecting flavor-related components in food. The current study sought to employ both GC–MS and GC–IMS to identify and characterize volatile compounds in Tibetan sheep meat. This was achieved by utilizing a combination of electronic tongue and electronic nose technologies, alongside an evaluation of the contributions of non-volatile substances to the overall flavor profile, to comprehensively assess the flavor characteristics of Tibetan sheep meat.

The primary objective was to analyze meat from Tibetan sheep raised in regions with saline-alkaline soils, focusing on four thermal processing methods: atmospheric-pressure cooking (AP), high-pressure cooking (HP), atmospheric-pressure high-pressure cooking (APHP), and high-pressure atmospheric-pressure cooking (HPAP). Sensory evaluation, alongside analyses of amino acids, nucleotides, and data from an electronic tongue, an electronic nose, GC–MS, and GC–IMS, was employed to investigate the impact of these four cooking methods on the flavor characteristics of Tibetan sheep meat.

## 2. Materials and Methods

### 2.1. Collection of Experimental Samples

The samples were collected from the Wayuxiang Kamei Duo breeding farm in Gonghe County, Hainan Prefecture, Qinghai Province. Samples of hind leg meat were collected from six-month-old Tibetan sheep standardized raised indoors, after which the samples were transported back to the laboratory and stored at −80 °C for future analysis. The nutritional composition of the feed used in the Tibetan sheep feeding regimen is presented in Table S1.

### 2.2. Experimental Methods

#### 2.2.1. Sample Preparation

The meat was cut into evenly sized pieces (each sample weighed approximately 500 g), cleaned with hot water, and drained for later use. For steaming at atmospheric pressure (AP), the meat chunks were placed in a steaming pot for 2 h, after which they were minced in a grinder. For steaming under high pressure (HP), the samples were placed in a reverse-pressure, high-temperature sterilization pot, steamed under high pressure for 2 h, and then minced. The atmospheric-pressure high-pressure (APHP) and high-pressure atmospheric-pressure (HPAP) methods were performed as described above. The detailed processing and cooking conditions are shown in Table 1. The pressure was maintained at around 0.1 MPa.

#### 2.2.2. Sensory Evaluation

The sensory evaluation criteria are shown in Table S2. The evaluation team consisted of 20 students undertaking food-related studies (10 males and 10 females) who were seated in a controlled room, with each member assigned an individual space where environmental conditions were maintained as consistently as possible. During the testing sessions, purified water and unsalted crackers were provided as palate cleansers. In each evaluation session, panelists randomly assessed and scored four groups of muscle samples. The sensory evaluation was conducted over a period of three weeks, with each session lasting 1.5–2 h. The evaluators conducted sensory evaluations of the Tibetan sheep meat in terms of five aspects, namely, appearance, odor, taste, texture, and overall acceptability.

#### 2.2.3. Electronic Nose Assessments

Individual samples (10 g) were placed in 100 mL beakers, which were sealed with a double layer of plastic wrap. Three parallel replicates were prepared for each sample and were allowed to stand at room temperature for 1 h before testing on the machine. For the direct headspace aspiration method, the injection needle was inserted directly into the sealed beaker containing the sample, and the electronic nose was used for measurement. The measurement conditions were as follows: sampling time, 1 s per group; self-cleaning time of the sensor, 80 s; sensor zeroing time, 5 s; sample preparation time, 5 s; injection flow rate, 400 mL/min; and sampling time for analysis, 80 s.

#### 2.2.4. Electronic Tongue Assessments

Thirty grams of the sample was weighed out and added to 150 mL of purified water. The material was then poured into a household food processor and stirred for 30 s, followed by centrifugation at 4000 rpm for 5 min and collection of the supernatant for testing. The abstract syntax tree (ASTREE) electronic tongue system was used to assess samples using a five-flavor sensor array, including five sensors for bitterness, sourness, saltiness, umami, and sweetness. The collection time for each sample was 120 s, with 6 repeated measurements, allowing 3 stable data measurements.

#### 2.2.5. Determination of Free Amino Acids

Samples stored at −80 °C were weighed, and 60 mg was then added to 150 μL of water for MP homogenization. After vortex mixing for 60 s, 800 μL of a methanol–acetonitrile solution (1:1, *v*/*v*) was added, followed by 50 μL of an internal standard mixture (50 μm 16 isotopes). The final mixture was vortex mixed for 60 s, and after low-temperature sonication, performed twice for 30 min each, it was left at 20 °C for 1 h to precipitate proteins. This was followed by a 20 min centrifugation at 14,000 rcf and 4 °C, with the resulting supernatant freeze-dried prior to storage at −80 °C [13]. An Agilent 1290 Infinity LC ultra-performance liquid chromatography system was finally used to separate the samples. The standard was placed in a 4 °C autosampler, with a column temperature of 35 °C. Mobile phase A: 25 mM ammonium formate with 0.1% formic acid in water; mobile phase B: 0.1% formic acid in acetonitrile, at a flow rate of 300 μL/min, and an injection volume of 2 μL. An AB SCIEX5500 QTRAP mass spectrometer (AB SCIEX Company, Boston, MA, USA) in positive ion mode was used for the mass spectrometry analysis.

#### 2.2.6. Determination of Fatty Acids

Modified by the method of Zhang et al. [14], this study employed the GC–MS approach. We weighed 50 mg of the sample and extracted the lipids using a chloroform–methanol (2:1, *v*/*v*) mixture. Prior to extraction, the sample was methylated with a sulfuric acid–methanol solution for 30 min. The mixture was then extracted with n-hexane and washed with 5 mL of pure water and 25 μL of methyl salicylate (internal standard). The supernatant was collected for further analysis. Separation of the sample was performed using an Agilent 19091S-433UI capillary column (Agilent Technologies, Inc., Santa Clara, CA, USA). The initial temperature was maintained at 40 °C for 5 min, followed by an increase to 220 °C at a rate of 10 °C/min, and held at 220 °C for 5 min. The carrier gas used in this study was helium, with a flow rate of 1.0 mL/min. An Agilent 5977B MSD mass spectrometer was used for mass spectrometry analysis.

#### 2.2.7. HS–GC–IMS Analysis

Sample processing and determination conditions: one gram of sheep was weighed out, placed in a 20 mL headspace vial, and incubated for 15 min. Three replicates of each sample were analyzed. Headspace injection conditions: incubation temperature, 50 °C; incubation time, 20 min; injection volume, 500 µL; non-diversion injection; hatching speed, 500 r/min; and injection needle temperature, 85 °C. The GC–IMS (Haineng Future Technology Group Co., Ltd., Jinan, China) conditions were as previously described [15].

#### 2.2.8. HS–GC–MS Analysis

According to the method described by Ahamed et al. [16] with modifications, 2.5 g of the sample was weighed and placed into a 15 mL headspace vial. A 20% NaCl saturated solution was added for salting-out, and the vial was sealed and labeled for analysis. The headspace conditions involved placing the sample on a magnetic stirrer at 60 °C for 30 min, followed by the insertion of a pre-aged (250 °C, 5 min) 75 μm CAR/PDMS manual extraction fiber into the headspace vial for adsorption over 30 min. The extraction fiber was then inserted into the GC–MS injection port and desorbed for 600 s. GC/MS analysis was performed using an Agilent 7890B gas chromatograph (Agilent Technologies, Santa Clara, CA, USA) equipped with an Agilent 5973C mass spectrometer and an autosampler (PAL, Agilent). Helium (99.999% purity) was used as the carrier gas at a flow rate of 1 mL/min, with the injection port temperature set at 250 °C. Separation of compounds was achieved using a Supelcowax-10 capillary column (60 m × 0.32 mm, 0.25 μm film thickness, Agilent). The initial oven temperature was set at 40 °C and held for 3 min. Subsequently, the temperature was increased at a rate of 4 °C/min to 120 °C, then at 8 °C/min to 220 °C, and finally at 20 °C/min to 250 °C, where it was maintained for 5 min.

#### 2.2.9. Taste Activity Values (TAV) and Relative Odor Activity Value (ROAV) Calculation

The taste activity value (TAV) was employed to evaluate the impact of free amino acids on the flavor of Tibetan lamb meat. The higher the TAV of a flavor compound, the greater its contribution to the overall flavor profile of the sample. The TAV was calculated as TAV = C_i_/T_i_, where C_i_ represents the concentration of the measured substance (mg/100 g), and Ti represents the taste threshold of the substance (mg/100 g) [17].

The relative odor activity value (ROVA) was calculated as ROAV = (C_A_/C_max_) × (T_max_/T_A_) × 100, where C_A_ represents the relative percentage content (%) of volatile component A, T_A_ represents the aroma threshold of compound A (μg/kg), and C_max_ and T_max_ indicate the relative percentage content (%) and aroma threshold (μg/kg), respectively, of the aroma components that contribute the most to the overall flavor of Tibetan sheep [18]. An ROAV > 1 indicates that a specific component contributes the most to the flavor of the sample; in other words, it represents the key characteristic flavor component, while 0.1 ≤ ROAV < 1 indicates a modifying effect on the flavor of the sample, and ROAV < 0.1 indicates that the component has no significant effect on the flavor of the sample. Within a certain range, the larger the ROAV, the greater the contribution of the substance to the overall flavor [19].

### 2.3. Data Processing

According to the principle of parallelism, all experiments were performed at least three times, and the results are expressed as mean ± standard deviation (SD). The data were preliminarily processed using Excel, after which SPSS software (version 26.0) was used for variance analysis, and Origin 2022 software was used for graphics. Orthogonal partial least squares discriminant analysis (OPLS-DA) was performed using SIMCA 14.1 software, calculating the variable in importance projection (VIP) of the predictor variables. Meanwhile, Pearson’s correlation analysis was performed to evaluate the relationships among the variables. A statistically significant correlation was defined as *p* < 0.05 with |r| > 0.50.

## 3. Results and Discussion

### 3.1. Effects of Different Steaming and Cooking Methods on the Sensory Evaluation of Tibetan Sheep Meat

**Figure 1:**
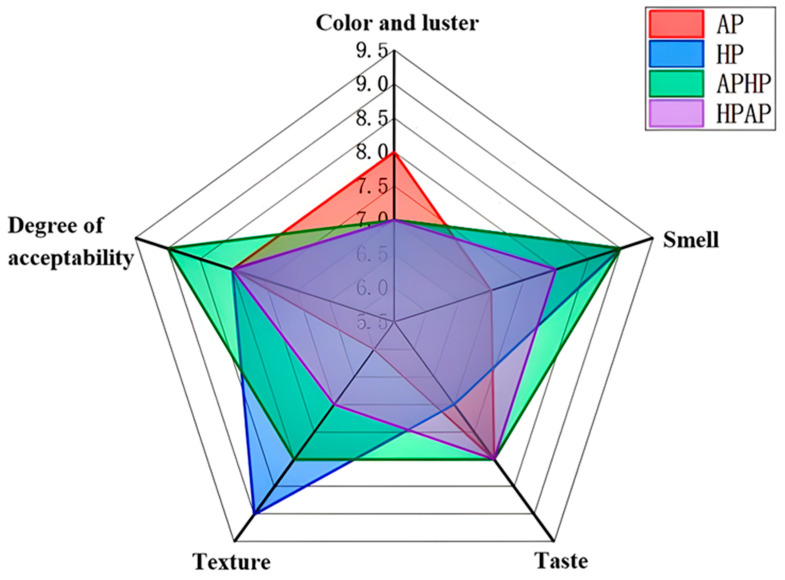
Sensory radar diagram of Tibetan sheep by different cooking methods. Note: AP refers to atmospheric pressure cooking, HP refers to high-pressure cooking, APHP refers to atmospheric pressure high-pressure cooking, and HPAP refers to high-pressure atmospheric pressure cooking; the same applies below.

The impact of various steaming and cooking techniques on the sensory evaluation of Tibetan sheep meat is presented in Figure 1 and Table S3. Sensory evaluation assessed five attributes of the cooked meat: color, odor, taste, texture, and overall acceptability. As illustrated in Figure 1, the aroma and acceptability of the Tibetan sheep meat in the APHP (atmospheric-pressure high-pressure) group were significantly higher compared to those prepared using alternative cooking methods (*p* < 0.05). Nevertheless, no significant difference was observed between the APHP and HP groups overall. Additionally, the sensory evaluation indicated that the atmospheric-pressure cooking method yielded the lowest scores. This may be due to the fact that certain flavor precursors in the sheep undergo a series of chemical reactions under high pressure and high temperature, resulting in the formation of various volatile compounds that enhance the tenderness, taste, and flavor of the cooked sheep.

### 3.2. The Effects of Different Steaming and Cooking Methods on the Odor Characteristics of Tibetan Sheep Meat

As depicted in Figure S1, an electronic nose system comprising ten sensors was utilized to differentiate the odor characteristics of Tibetan sheep meat subjected to various cooking methods. The analysis of sensor response curves indicates that the sensors exhibited robust responses, reflecting the distinctive odor profile of the meat. Notably, several sensors demonstrated significant responses, with sensor R2 displaying the highest response value among sensors R6, R7, R8, and R9. In contrast, sensors R1, R4, R5, and R10 also exhibited notable responses, while sensors R1 and R3 showed minimal variation in response values throughout the process, maintaining values closest to 1. From the perspective of sensor response, sensor R7 exhibited the highest response value to meat in the AP group, whereas sensor R6 demonstrated a stronger response to meat in the APHP, HP, and HPAP groups throughout the testing process. The sensor responses exhibited similar trends across the APHP, HP, and HPAP groups.

**Figure 2:**
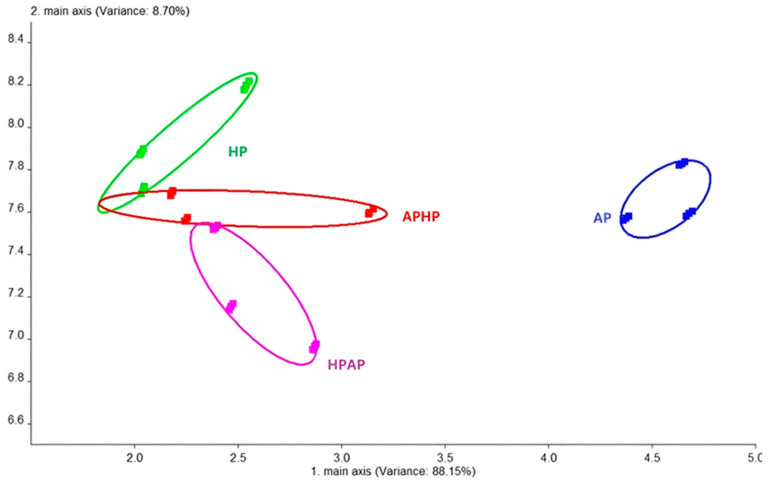
Principal component analysis of electronic nose results for cooked Tibetan sheep meat under different cooking methods. Note: AP refers to atmospheric pressure cooking, HP refers to high-pressure cooking, APHP refers to atmospheric pressure high-pressure cooking, and HPAP refers to high-pressure atmospheric pressure cooking; the same applies below.

Furthermore, the results of the principal component analysis (PCA) for the Tibetan sheep meat samples subjected to four distinct cooking methods are presented in Figure 2. The analysis revealed that the first principal component accounted for 88.15% of the variance, while the second component accounted for 8.70%, cumulatively explaining 96.85% of the total variance. These results suggest significant differences in the overall odor profile of Tibetan sheep meat based on the cooking method used. Notably, the meat from the AP group showed substantial separation from the other three groups, primarily along the first principal component. In contrast, the HP, APHP, and HPAP groups exhibited relatively minor intergroup distances, indicating a degree of similarity in their overall odor profiles, which aligns with the sensory evaluation results. Although some variation is observed in the second principal component, the primary differences are predominantly associated with the first principal component.

### 3.3. The Effects of Different Steaming and Cooking Methods on the Taste Characteristics of Tibetan Sheep Meat

The taste characteristics of Tibetan sheep meat prepared using different cooking methods were assessed using an electronic tongue analyzer equipped with five sensors. Principal component analysis (PCA) was initially employed to reduce the dimensionality of the raw data (Figure S2). It was found that the four groups were located in different quadrants, indicating significant differences in the taste of the meat following the AP, HP, APHP, and HPAP groups. The contribution of the first principal component was 86.28%, while that of the second principal component was 12.38%, indicating that the differences were primarily associated with the first principal component.

**Figure 3:**
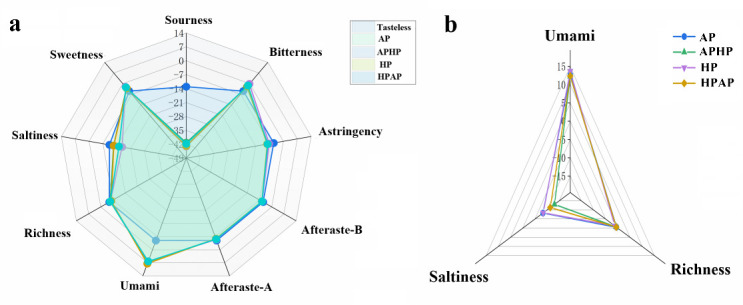
Radar map (**a**) and umami, salty, and richness radar map (**b**) of Tibetan sheep based on RefSol reference solution. Note: AP refers to atmospheric pressure cooking, HP refers to high-pressure cooking, APHP refers to atmospheric pressure high-pressure cooking, and HPAP refers to high-pressure atmospheric pressure cooking; the same applies below.

As illustrated in Figure 3a, the term “tasteless” denotes the outcome for the reference solution, which consisted of KCl and tartaric acid, with sour and salty taste values of −13 and −6, respectively. Taste values of samples that fall below the “tasteless” reference suggest an absence of a particular taste, whereas values above indicate the presence of that taste. From Figure 3a,b, it is evident that the Tibetan sheep meat exhibited lower values than the “tasteless” benchmark for sour taste, astringency, bitter aftertaste, astringency aftertaste, richness, and salty taste, while the values for bitterness, umami, and sweetness were higher. The sweetness and bitterness values in the APHP and HPAP groups exceeded those in the AP and HP groups, whereas the saltiness and umami values were lower. Notably, the APHP group demonstrated the highest levels of sweetness and bitterness but the lowest levels of saltiness and umami. In contrast, the HPAP group also exhibited elevated sweetness and significantly reduced bitterness compared to APHP, along with diminished salty and umami tastes. HP showed the greatest freshness but the least richness and sweetness, consistent with the findings on flavor-associated amino acids, and the AP group also had a slightly lower sweetness value, the lowest value for bitterness, and the highest values for saltiness and richness.

### 3.4. The Effects of Different Cooking Methods on the Free Amino Acid Contents of Tibetan Sheep Meat

As presented in Table 2, the analysis of amino acid content in Tibetan sheep meat subjected to various cooking methods reveals that the high-pressure (HP) group exhibited significantly elevated levels of umami amino acids, essential amino acids, and total free amino acids compared to other cooking methods (*p* < 0.05). Conversely, the amino acid content following atmospheric-pressure high-pressure (APHP) cooking was generally the lowest. Notably, glutamic acid demonstrated the highest taste activity value (TAV) among the umami amino acids, signifying its primary contribution to the umami flavor of Tibetan sheep meat. Additionally, the amino acids alanine (Ala), glycine (Gly), arginine (Arg), and methionine (Met), which are associated with sweetness, were identified as the main contributors to the sweet flavor due to their elevated TAVs. Furthermore, the bitter amino acids histidine (His), isoleucine (Ile), and phenylalanine (Phe) exhibited higher TAVs, thereby serving as the principal contributors to the bitter flavor of Tibetan sheep meat. The TAVs for umami amino acids in the atmospheric-pressure (AP), APHP, HP, and high-pressure atmospheric-pressure (HPAP) groups were 37.71, 10.82, 41.14, and 19.66, respectively. Similarly, the TAVs for sweet amino acids were 44.5, 17.98, 51.41, and 33.13, respectively. The study revealed that the concentrations and taste activity values (TAV) of fresh, sweet, and bitter amino acids in meat subjected to high-pressure cooking were significantly elevated compared to the other three treatment groups. Consequently, Tibetan sheep meat predominantly exhibited sweet flavors, complemented by fresh flavors. This finding aligns with Xiang et al.’s analysis of sheep flavor profiles, which identified sweet amino acids as the most prevalent [20]. The comprehensive analysis of flavor-associated amino acids suggests that high-pressure steaming is the optimal thermal processing method for Tibetan sheep meat, corroborating the results of the sensory evaluation.

### 3.5. The Effects of Different Steaming and Cooking Methods on Fatty Acids in Tibetan Sheep Meat

The comparison of the fatty acid contents of Tibetan sheep meat after different steaming methods is shown in Table 3. A total of 40 fatty acids were detected from the four groups of samples. As indicated in Table 3, the fatty acid content of Tibetan sheep meat differed significantly (*p* < 0.05) among different cooking-method groups. In terms of the SFA, MUFA, PUFA, and n-6/n-3 contents, the ranking of the groups was AP > APHP > HPAP > HP (*p* < 0.05), with the AP group showing markedly higher levels of C12:0, C14:0, C14:1n5, C16:0, C18:0, C18:2n6, and C18:3n6 than the other treatment groups (*p* < 0.05). The concentration of C24:1n9 was higher in HP, while the levels of C20:4n6, C22:6n3, C22:5n3, and C22:5n6 were higher in APHP. In addition, the concentrations of C22:1n9 and C22:4n6 were higher in the HPAP group. However, the presence of C4:0 and C15:1 acid was not detected in the four samples.

Ruminant meat represents an important source of fatty acids in the human diet [21]. These fatty acids not only influence the quality of the meat but also modulate the levels of low-density lipoprotein (LDL) cholesterol in the bloodstream [22]. In this study, a total of 40 fatty acids were detected in Tibetan sheep meat after different methods of cooking, with the diverse types and concentrations of fatty acids enhancing the meat’s flavor. It has been established that saturated fatty acids (SFAs) can elevate LDL cholesterol levels [23], potentially contributing to cardiovascular disease; however, not all SFAs result in increased cholesterol. Notably, stearic acid (C18:0) and palmitic acid (C16:0), the predominant SFAs in meat, exhibit different effects: stearic acid (C18:0) does not elevate cholesterol levels and may offer protective effects against certain cancers [24], whereas palmitic acid (C16:0) has a minimal impact on blood lipid levels [25].

Furthermore, fatty acids, particularly unsaturated fatty acids, play a crucial role in the flavor profile of meat [26]. Monounsaturated fatty acids (MUFAs) are associated with various physiological benefits, including cardioprotection, the mitigation of hypoglycemia and cholesterol levels, and the prevention of memory loss [27,28]. Notably, oleic acid (C18:1N9), a predominant MUFA, is known to enhance immune function and exert protective effects against cancer and inflammatory diseases [29]. Polyunsaturated fatty acids (PUFAs) are involved in the regulation of lipid metabolism and immunity, possess anticancer properties, aid in the prevention and treatment of cardiovascular diseases, support growth and development, and influence gene expression [30]. In this study, stearic acid (C18:0), palmitic acid (C16:0), and oleic acid (C18:1N9) were identified as the primary fatty acids present in Tibetan sheep meat, corroborating previous research findings [31]. Furthermore, the study revealed that the concentrations of saturated fatty acids (SFAs), monounsaturated fatty acids (MUFAs), and polyunsaturated fatty acids (PUFAs) followed the order: AP group > APHP group > HPAP group > HP group. This pattern may be attributed to the oxidative decomposition of fatty acids resulting from prolonged exposure to high temperature and pressure, which enhances the levels of volatile components. These findings are corroborated by the results obtained from gas chromatography–mass spectrometry (GC–MS) and gas chromatography–ion mobility spectrometry (GC–IMS).

### 3.6. GC–IMS Analysis of Volatile Constituents of Tibetan Sheep Meat After Different Steaming Methods

#### 3.6.1. GC–IMS Profiling of Tibetan Sheep Cooked with Different Steaming Methods

**Figure 4:**
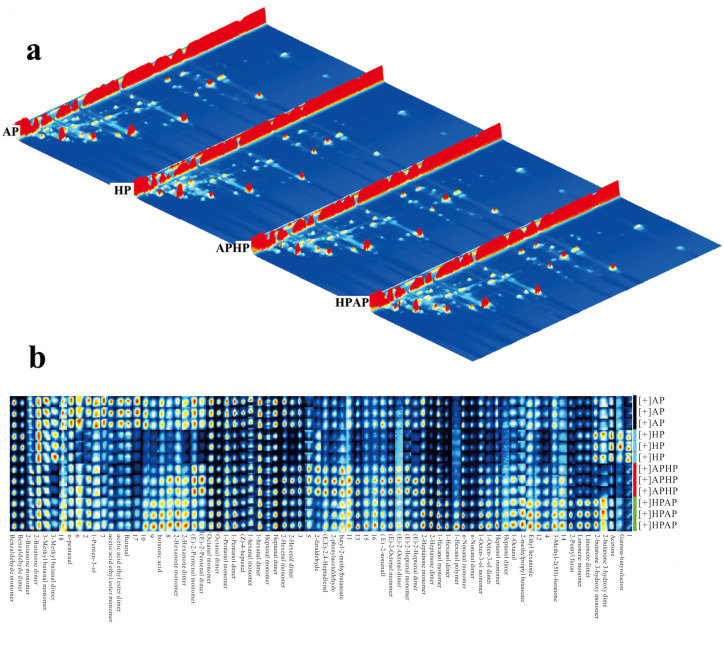
3D spectrum (**a**) and fingerprint (**b**) of the volatile components of Tibetan sheep by different cooking methods. Note: AP refers to atmospheric pressure cooking, HP refers to high-pressure cooking, APHP refers to atmospheric pressure high-pressure cooking, and HPAP refers to high-pressure atmospheric pressure cooking; the same applies below.

The three-dimensional GC–IMS spectra of Tibetan lamb meat prepared using various cooking methods are depicted in Figure 4a. In this figure, the *X*-axis denotes the ion migration time, the *Y*-axis indicates the GC signal retention time, and the *Z*-axis represents the intensity of the signal peaks. Peaks located to the right of the RIP peak correspond to volatile compounds, with the color gradient from blue to red signifying the peak intensity and concentration, where darker hues represent higher values; the baseline color is blue. The figure illustrates that, while the overall distribution of volatile compounds is generally similar across the different cooking-method groups, notable variations exist in the location and intensity of specific characteristic peaks.

A detailed comparison of volatile compounds across various cooking methods for Tibetan sheep meat was conducted using fingerprinting analysis, as illustrated in Figure 4b. Each row in the figure represents the signal peaks selected from an individual sample, while each column corresponds to the signal peaks of the same volatile organic compound across different samples. This visualization allows for a comprehensive assessment of the volatile organic compounds present in each sample and highlights the differences between them. Each peak on the right side of the reaction ion peak represents a volatile compound, and the color represents the peak intensity of the substance. From blue to red, the darker the color, the greater the peak intensity, that is, the higher the concentration. The comparative analysis revealed that the volatile profiles of the APHP and HPAP samples were more similar. Notably, the compounds in region A of the figure, such as valeraldehyde, 1-penten-3-ol, ethyl acetate, butyraldehyde, and 3-methyl n-butyraldehyde, exhibited higher concentrations in the AP samples.

#### 3.6.2. Qualitative and Quantitative Analysis (GC–IMS) of Volatile Constituents of Tibetan Sheep in Different Cooking Methods

To investigate the impact of various cooking methods on the volatile flavor compounds in Tibetan lamb meat, gas chromatography–ion mobility spectrometry (GC–IMS) was employed to statistically analyze the volatile compounds following different cooking techniques, as detailed in Table S4. The analysis identified 76 volatile compounds across the four cooking method groups. Among these, 58 compounds were characterized, including both dimers and monomers, which could not be achieved using gas chromatography–mass spectrometry (GC–MS), as reported by Guo et al. [32]. The identified compounds comprised twenty-seven aldehydes, eleven alcohols, ten ketones, six esters, two olefins, one heterocyclic compound, and one type of acid.

As shown in Table S4, the volatile flavor compounds exhibited significant intra-group variations. The composition and content of aldehydes in the AP group were significantly higher than those of other volatile compounds. Notably, octanal monomer, heptaldehyde dimer, and 1-hexanal dimer were the predominant contributors, imparting fatty, citrus-like, grassy, and buttery notes to Tibetan sheep meat. Furthermore, both the composition and content of alcohols and ketones were notably elevated. Compounds including 1-Hexanol monomer, 1-octen-3-ol monomer, 1-pentanol dimer, 2-butanone monomer, and 2-heptanone monomer contributed distinct floral, balsamic, and soap-like aroma characteristics. Meanwhile, the HP, APHP, and HPAP groups also exhibited high levels of aldehydes. Key flavor compounds such as octanal monomer, Heptaldehyde dimer, Heptaldehyde monomer, and 1-Hexanal dimer contributed fatty, citrus, and buttery flavors to the high-pressure-cooked Tibetan sheep meat. In the APHP group, compounds including n-Pentanal, octanal dimer, octanal monomer, and heptaldehyde dimer were responsible for almond, malty, and fatty characteristics. Furthermore, the HPAP group was characterized by mushroom and fatty flavors, primarily derived from volatile compounds such as heptaldehyde dimer, 1-hexanal dimer, 1-octen-3-ol monomer, and 1-penten-3-ol monomer.

As presented in Table S4, notably, aldehydes were more prevalent in the atmospheric-pressure (AP) group, whereas alcohols, ketones, and esters were significantly more abundant in the high-pressure atmospheric-pressure (HPAP) group compared to the other groups. Conversely, the concentration of volatile compounds was generally lower in the high-pressure (HP) group, likely due to the loss of volatiles from Tibetan sheep meat after prolonged exposure to high temperature and pressure.

Furthermore, aldehydes emerged as the most frequently detected volatile compounds across all four samples of Tibetan sheep. Aldehydes are recognized as significant contributors to the flavor profile of meat products, attributed to their low odor thresholds and the overlapping nature of their aromas [33]. These compounds are essential volatile constituents in food products, primarily arising from the oxidation of fats and amino acids during the Strecker degradation reaction [34]. As illustrated in Table S4, seven volatile compounds exhibited significant differences (*p* < 0.05) across the four samples, with the majority being aldehydes. These include (E)-2-nonenal, (E)-2-octenal monomer, (E)-2-octenal dimer, (E, E)-2-4-heptadienal, (Z)-4-heptenal, (E)-2-pentenal monomer, (E)-2-pentenal dimer, (E)-2-heptenal monomer, (E)-2-heptenal dimer, nonenal dimer, renal monomer, pentenal, octenal dimer, octanal monomer, and 2-hexenal dimer. Specifically, hexanal and heptanal are oxidation products of linoleic acid and arachidonic acid, respectively [35], whereas octanal and nonanal result from the oxidation of oleic acid [36]. Notably, hexanal, heptanal, octanal, and nonanal are characterized by greenish and fatty odors.

The study identified the predominant volatile compounds as 2-hexenal monomer, 2-phenylacetaldehyde, isovaleraldehyde dimer, isovaleraldehyde monomer, benzaldehyde dimer, benzaldehyde monomer, n-butyraldehyde, heptanal dimer, heptanal monomer, 1-hexanal dimer, 1-hexanal monomer, and 2-furaldehyde. Notably, 2-hexenal dimer, 2-hexenal monomer, heptanal dimer, and heptanal monomer were associated with fatty flavors and putrid odors and were present in significantly lower concentrations in high-pressure-cooked Tibetan sheep compared to the other three cooking methods. In contrast, the concentrations of (Z)-4-heptadienal, glutaraldehyde, and hexanal were relatively elevated in atmospheric-pressure-steamed Tibetan sheep meat, imparting flavors reminiscent of cookies, cream, and butter, along with a pungent taste. Furthermore, the concentrations of 2-phenylacetaldehyde, (E)-2-octenal, and (E)-2-nonenal were significantly higher in meat prepared using an atmospheric-pressure autoclave and high-pressure atmospheric-pressure steaming, resulting in flavors characteristic of hawthorn and honey, as well as a sweet taste.

Alcohols, which possess higher detection thresholds than aldehydes [37], contributed minimally to the overall flavor profile of the samples, although they exhibited a synergistic effect on the overall flavor [38]. As illustrated in Table S4, the concentrations of 1-pentanol and 1-penten-3-ol were notably elevated in the AP samples compared to those prepared using other cooking methods, imparting a tangy, buttery, and pungent aroma. The levels of 1-hexanol dimer, 1-hexanol monomer, heptanol dimer, heptanol monomer, 1-octanol, and 1-octen-3-ol were generally higher in the meat from the HPAP group, whereas the HP group exhibited the lowest concentrations of these compounds. Furthermore, elevated levels of 1-octen-3-ol, alongside higher relative odor value activity (ROVA) and Variable Importance in Projection (VIP) values, were identified as playing a significant role in the flavor development of Tibetan sheep across all four sample groups [20].

Esters, formed through the esterification of alcohols and carboxylic acids in Tibetan sheep, typically impart a fruity flavor. Previous research has demonstrated that short-chain esters are associated with fruity aromas, while long-chain esters contribute to a fatty flavor profile [39]. In the Tibetan sheep meat subjected to the four cooking methods, only short-chain esters were identified, predominantly including ethyl acetate D, ethyl acetate M, butyl 2-methylbutyrate, ethyl caproate, and butyrolactone. Notably, butyrolactone was present in relatively high concentrations in the meat from the HP group, imparting a sweet flavor. As a lactone, butyrolactone contributes a long-lasting flavor and significantly influences the overall taste profile of the sheep meat. Conversely, the levels of ethyl acetate D and ethyl acetate M were elevated in the meat from the AP group compared to the other samples, imparting a pineapple-like flavor. Furthermore, ketones, which can arise from the Maillard reaction between sugars and proteins or from the β-oxidation of fatty acids [40], were also detected. The primary ketones identified included 2-butanone D, 2-butanone M, 2-heptanone D, 2-heptanone M, 2-hexanone D, 2-hexanone M, and acetone, which contributed etheric, soapy, and pungent flavors, serving as the main contributors to the distinctive aroma of Tibetan sheep meat.

In the Tibetan sheep meat subjected to the four cooking methods, only short-chain esters were identified, predominantly including ethyl acetate D, ethyl acetate M, butyl 2-methylbutyrate, ethyl caproate, and butyrolactone. Notably, butyrolactone was present in relatively high concentrations in the meat from the HP group, imparting a sweet flavor. As a lactone, butyrolactone contributes a long-lasting flavor and significantly influences the overall taste profile of the sheep meat. Conversely, the levels of ethyl acetate D and ethyl acetate M were elevated in the meat from the AP group compared to the other samples, imparting a pineapple-like flavor. Furthermore, ketones, which can arise from the Maillard reaction between sugars and proteins or from the β-oxidation of fatty acids [40], were also detected. The primary ketones identified included 2-butanone D, 2-butanone M, 2-heptanone D, 2-heptanone M, 2-hexanone D, 2-hexanone M, and acetone, which contributed etheric, soapy, and pungent flavors, serving as the main contributors to the distinctive aroma of Tibetan sheep meat.

The ketone concentrations in the HPAP group were markedly elevated compared to those in the other groups. This is likely attributable to the sequential high-pressure heating followed by atmospheric pressure, which facilitates the release of ketone volatiles from the Tibetan sheep meat. Among the four cooking method groups, the butyric acid content was highest in the samples cooked under atmospheric pressure, and those subjected to high pressure followed by ordinary pressure. This was followed by the atmospheric-pressure/ordinary-pressure cooking method, while the lowest levels were observed in the high-pressure-cooked samples. Consequently, significant variations in the volatile compound contents were observed across the samples subjected to different heating methods. These differences may be ascribed to the distinct heat transfer mechanisms, as well as variations in heating duration, temperature, and pressure associated with the different steaming conditions [41].

#### 3.6.3. PCA Analysis (GC–IMS) of Volatile Constituents of Tibetan Sheep Meat After Different Methods of Cooking

**Figure 5:**
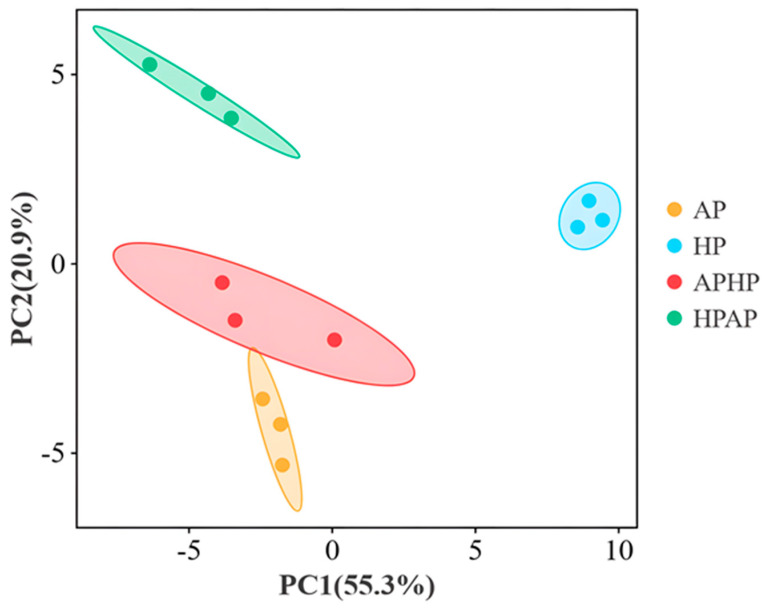
PCA analysis of volatile components of Tibetan sheep by different cooking methods. Note: AP refers to atmospheric pressure cooking, HP refers to high-pressure cooking, APHP refers to atmospheric pressure high-pressure cooking, and HPAP refers to high-pressure atmospheric pressure cooking; the same applies below.

The principal component analysis (PCA) of volatile compounds in Tibetan sheep meat subjected to various cooking methods is illustrated in Figure 5. The figure reveals that the first principal component (PC1) accounts for 55.3% of the variance, while the second principal component (PC2) contributes 20.9%, culminating in a cumulative variance contribution of 76.2%. This suggests significant differences in the volatile compounds of Tibetan sheep meat across different cooking methods (*p* < 0.05). Notably, there is a clear separation between the HP group and the AP, APHP, and HPAP groups, indicating a more pronounced difference between HP and the other cooking methods. Conversely, the proximity of the AP, APHP, and HPAP groups suggests a similarity in the concentrations of volatile compounds among these three treatments.

#### 3.6.4. OPLS–DA Analysis (GC–IMS) of Volatile Constituents of Tibetan Sheep Meat After Different Methods of Cooking

**Figure 6:**
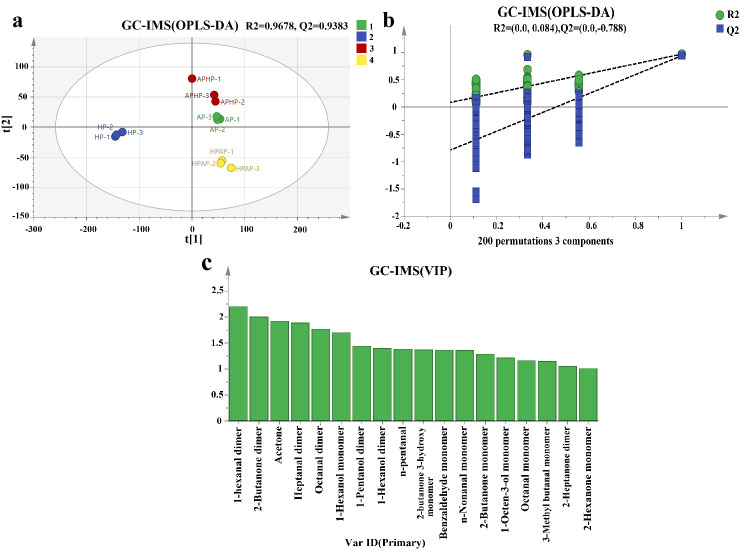
Fractional scatter plot (**a**), displacement test result (**b**), and VIP (**c**) of volatile OPLS–DA of Tibetan sheep by different cooking methods (*p* < 0.05, VIP > 1). Note: t [1] and t [2] are principal component dimensions adjusted with coefficients.

Figure 6 presents the analysis of flavor substances in Tibetan lamb, cooked using different methods, as assessed by the orthogonal partial least squares discriminant analysis (OPLS–DA) model. As depicted in Figure 6a, the four treatment groups are distinctly separated within the model, underscoring significant differences in flavor substances among the samples (*p* < 0.05). The AP and APHP groups, however, are in close proximity, indicating a similarity in their flavor profiles. In addition, the model’s R^2^ = 0.968 and Q^2^ = 0.938 were close to 1, indicating that the model was accurate and had good predictive ability. The results of the 200-permutation test are shown in Figure 6b, where the horizontal coordinate represents the retention of the samples, and the R^2^ and Q^2^ of the original model are seen at 1.0. This verified that both R^2^ (0.0998) and Q^2^ (−0.781) are below the retention value of 1 and that the intercept of the regression line of the model’s Q^2^ with the horizontal coordinate is negative, which indicates that the model is free of overfitting and is stable and reliable.

The variable influence on projection (VIP) value was employed to evaluate the abundance patterns of various metabolites and to determine the intensity and explanatory power of their influence on the categorical differentiation of each sample group. A higher VIP value indicates a greater contribution of the parameter to the flavor profile of Tibetan sheep meat. As illustrated in Figure 6c and detailed in Table S5, a VIP threshold of >1.5 was utilized to identify differentially abundant compounds, thereby highlighting key flavor-associated substances such as hexanal dimer, 2-butanone dimer, acetone, heptanal dimer, octanal dimer, and 1-hexanol monomer. Notably, hexanal dimer and 2-butanone dimer exhibited VIP values exceeding 2, indicating a more significant contribution to the flavor of Tibetan sheep meat. The concentrations of heptanal dimer and hexanal dimer were elevated in the AP group, whereas acetone and 2-butanone dimer were more abundant in Tibetan sheep meat from the HP group. Additionally, the levels of 1-hexanol monomer and octanal dimer were higher in the HPAP group. Thus, these volatile compounds play a key role in the flavor of Tibetan sheep after different methods of cooking.

#### 3.6.5. Identification of Key Volatile Flavor Substances (GC–IMS)

The key flavor compounds and relative odor value activity (ROVA) metrics of Tibetan sheep meat subjected to various cooking methods are presented in Table 4. ROVA values effectively illustrate the contribution of volatile compounds to the overall flavor profile of Tibetan sheep meat across different treatment groups, with higher ROVA values signifying a more substantial impact on the sample’s overall flavor. Compounds with ROVA values of 1 or greater were identified as the primary contributors to the flavor profile, whereas those with ROVA values between 0.1 and 1 were considered to be flavor-modifying substances that influence the overall flavor of the samples. As depicted in Table 4, the relative concentration of 1-hexanal was notably higher compared to other compounds, with a threshold value of 0.005 mg/kg, indicating its significant impact on the flavor profile of Tibetan sheep meat; consequently, its ROVA value was assigned as 100. Furthermore, Table 4 reveals that 15 volatile components exhibited ROVA values of 1 or greater, including (E)-2-nonenal, (Z)-4-heptenal, Isovaleraldehyde (CH20)3, (E)-2-octenal, 1-octanol, 2-butanone-3-hydroxy, 2-hexenal, 2-methylpropylbutyrate, 2-pentylfuran, 2-phenylacetaldehyde, butyraldehyde, hexyl hexanoate, heptanol, limonene, and glutaraldehyde, which thus represent key volatile compounds in Tibetan sheep meat.

Overall, among these volatile compounds with ROVA values >1, most exhibited higher ROVA values in high-pressure-treated Tibetan lamb meat. This indicates that, under a fixed duration, Tibetan lamb meat subjected to high temperature and high pressure releases volatile compounds with higher ROVA values and superior flavor characteristics. Particularly, among the key volatile compounds, the ROVA of isovaleraldehyde (CH20)3—associated with malt—and ethyl hexanoate—linked to apple peel and fruit and butanal—exhibited a significant increase in the HP group compared to the AP group. Meanwhile, the ROVA values for 1-octanol (characterized by green, nut, and fat notes), 1-octen-3-ol (mushroom), 2-pentylfuran (mung bean, butter), 2-phenylglycolal (hawthorn, honey, sweet), and limonene (lemon, orange) were notably elevated in the HP, APHP, and HPAP groups. Additionally, compared to the HP group, (E)-2-octenal, 1-octanol, 1-octen-3-ol, 2-pentylfuran, and 2-phenylacetaldehyde exhibited higher ROVA values in both HPAP and APHP groups. This suggests that prolonged high-pressure treatment may reduce the ROVA values of certain favorable flavor compounds. Conversely, the ROVA values of 2-hexenal (fat, rancid) and valeraldehyde (almond, malt, spicy) were significantly higher in the APHP group compared to the other treatment groups, potentially serving as the primary contributors to the off-flavors associated with atmospheric-pressure-cooked Tibetan sheep meat. Among the modifying compounds, 2-butanone, 2-heptanone, and 2-hexanone, which possess irritating odors, showed a significantly higher ROVA in the AP group than in the HP group, yet lower than in the APHP and HPAP groups.

### 3.7. GC–MS Analysis of Volatile Components of Tibetan Sheep Meat After Different Methods of Cooking

#### 3.7.1. PCA Plot of Volatile Components Identified by GC–MS

**Figure 7:**
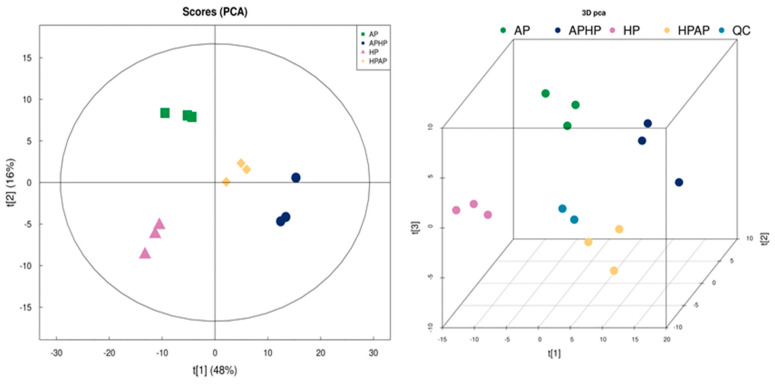
PCA analysis of volatile components in Tibetan sheep by different cooking methods.

To evaluate the variations in volatile compounds present in Tibetan sheep meat subjected to different cooking methods, the compounds identified through gas chromatography–mass spectrometry (GC–MS) were statistically analyzed using principal component analysis (PCA). As illustrated in Figure 7, the first principal component accounted for 48% of the variance, while the second principal component contributed 16%, culminating in a cumulative variance contribution of 64% from these two components. The separation between the HP group and the AP, APHP, and HPAP groups was considerable, indicating significant differences in volatile compounds between the HP group and the other groups (*p* < 0.05). In contrast, the proximity between the APHP and HPAP groups suggested similarities in their volatile compound profiles, corroborating the findings from the GC–IMS analysis.

#### 3.7.2. Qualitative and Quantitative Analysis (GC–MS) of the Aroma Components of Tibetan Sheep After Different Methods of Cooking

To further elucidate the impact of different cooking methods on the aroma components of Tibetan sheep meat, the volatile compounds were subjected to both qualitative and quantitative analysis via GC–MS. As detailed in Table S6, a total of 365 aroma compounds were identified across the four groups, among which alcohols, esters, aldehydes, and ketones were the main flavor substances in Tibetan sheep, consistent with previous results [20].

Overall, the HP group exhibited a significantly greater abundance of aroma-associated compounds (*p* < 0.05) compared to the other three treatment groups, potentially due to the reduced fatty acid content in the autoclaved Tibetan sheep. Notably, compounds such as 1-octen-3-ol, 3-methyl-1-butanol, 2-methyl-1-butanol, 2-octanol, hexyl acetate, 5-pentanedihydro-2(3H)-furanone, 3-pentanone, 2-nonanone, (E,E)-2,4-heptadienal, 4-ethylbenzaldehyde, and hexanal contributed to fruity, sweet, nutty, and caramelized flavor profiles in the meat. The relative concentrations of aldehydes were significantly elevated (*p* < 0.05) in the AP and HP groups compared to the APHP and HPAP groups. This was particularly evident for aldehydes such as 1-hexenal, (Z)-4-heptenal, heptanal, renal, (E,E)-2,4-heptadienal, (E,Z)-2,4-decadienal, 4-ethylbenzaldehyde, 2-undecenal, (E)-2-octenal, 3-furaldehyde, 3-methyl butyraldehyde, (E)-2-heptenal, (Z)-2-decenal, hexanal, and (Z)-6-nonenal, with heptanal, renal, and (E)-2-heptenal exhibiting higher relative concentrations in the HP group (*p* < 0.05). Furthermore, alcohols were the most frequently detected substances across the four groups of Tibetan sheep, with significantly higher levels (*p* < 0.05) observed in the AP and HP groups. Additionally, a variety of heterocyclic compounds, including pyrrole, pyrazine, and furan, were identified, particularly when compared to GC–IMS. Notably, dimethyl pyrazine, which imparts a barbecue flavor to Tibetan sheep, was found in higher concentrations in the HP, APHP, and HPAP groups, suggesting that high-pressure cooking facilitates the formation of heterocyclic compounds [42]. Moreover, sulfur-containing compounds such as dimethyl disulfide and trimethyl trisulfide were detected. These compounds have been shown to contribute to a sulfide odor and a fresh onion aroma, respectively [43].

As shown in Table S6, the composition and content of volatile compounds exhibited significant differences within the groups (*p* < 0.05). In the AP group, the composition and content of alcohols were significantly higher than those of other compounds (*p* < 0.05). Particularly, volatile flavor substances such as isopropyl alcohol, 1-hexanol, 1-octen-3-ol, 3-methyl-1-butanol, and ethanol imparted mushroom, floral, fruity, and alcoholic notes to the AP group. Secondly, although the variety of esters was relatively abundant, the total content of ketones was notably higher. In the APHP group, the composition and content of alcohols were also elevated, with flavor compounds such as 3-octanol, ethanol, 3-methyl-1-butanol, α,α,4-Trimethylbenzyl alcohol, and 1-hexanol contributing nutty, mushroom, alcoholic, and rose-like aromas. Additionally, the content of ketones was relatively high, with 3-octanone and 1-(1H-pyrrol-2-yl)-ethanone imparting cheesy, fruity, and roasted notes to the APHP group. In the HP group, the diversity of alcohols was considerable, but the content of ketones was significantly higher than that of other compounds (*p* < 0.05), particularly 1-(1H-pyrrol-2-yl)-ethanone, which conferred nutty and caramel-like aromas to the HP group. In the HPAP group, the composition and content of alcohols were significantly higher than those of other compounds (*p* < 0.05), with isopropyl alcohol and 1-hexanol contributing mushroom and floral notes. Furthermore, the content of esters was relatively high, and compounds such as ethyl 3-methylbutyrate and methyl 2-methyl-2-butenoate endowed the HPAP group with a strong fruity aroma.

#### 3.7.3. OPLS–DA Determination of Volatile Components of Tibetan Sheep Meat After Different Methods of Cooking

**Figure 8:**
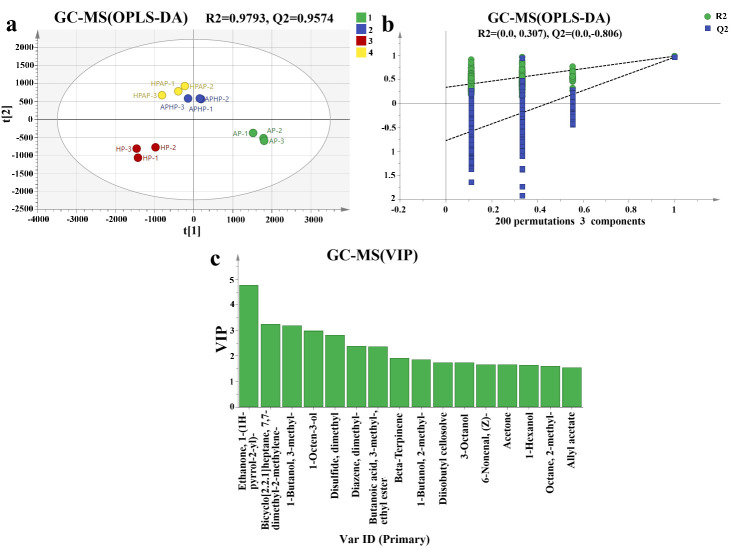
Score plot (**a**), replacement test plot (**b**), and VIP plot (**c**) of volatile constituents OPLS–DA of Tibetan sheep by different cooking methods (VIP > 1.5, *p* < 0.05). Note: t [1] and t [2] are principal component dimensions adjusted with coefficients.

OPLS–DA, a supervised discriminant analysis method [44], evaluates model accuracy and reliability using R^2^ and Q^2^ metrics. R^2^X and R^2^Y represent the explanation rates for the X and Y matrices, while Q^2^ indicates predictive ability. Values above 0.5 are typical, with values closer to 1 signifying stronger predictive power. Volatiles with *p* < 0.05 were analyzed using OPLS–DA and ROVA calculations. Figure 8a shows distinct separation among four treatment groups, with the HP group notably different in flavor substances (*p* < 0.05). To avoid overfitting, a replacement test was conducted, as shown in Figure 8b. Results from 200 tests indicated lower values on the left compared to the original data, and the Q^2^ line intersected the *Y*-axis below 0, confirming no overfitting. In addition, R^2^ = 0.9817 and Q^2^ = 0.9612, with both R^2^ and Q^2^ greater than 0.9, which indicates that the model has good explanatory and predictive ability.

As illustrated in Figure 8c and Table S7, a criterion of VIP > 1.5 was employed to identify differential compounds. A total of 20 flavor compounds were identified, compounds such as 1-(1H-pyrrol-2-yl)-ethanone, 7,7-dimethyl-2-methylenebicyclo[2.2.1]heptane, dimethyldisulfide, and dimethyldiazene were found in higher relative concentrations in the HP group, while the levels of ethyl 3-methylbutanoate were higher in the HPAP group. Additionally, 1-octen-3-ol and 3-methyl-1-butanol exhibited relatively high concentrations in the AP group.

#### 3.7.4. Identification of Key Volatile Flavor Substances (GC–MS)

To identify key flavor compounds in Tibetan sheep meat after various cooking methods, ROVA values were calculated for volatile compounds with *p* < 0.05. Compounds with ROVA > 1 were listed in Table 5. Notably, 3-methyl-1-butanol, with a threshold of 0.25 mg/kg and a ROVA set at 100, significantly contributes a malty and caramelized flavor. Overall, GC–MS and GC–IMS results demonstrated consistency, with most flavor compounds exhibiting ROVA values >1 showing higher levels in the HP, APHP, and AP groups, including S-methyl propylthioate, trimethylpyrazine, 2-methyl-1-butanol, 2-octanol, dimethyl disulfide, 3-methylphenol, (-)-carvone, 1-hexanol, 5-pentyl dihydro-2(3H)-furanone, 5-ethyldihydro-2(3H)-furanone, and 1-octen-3-ol. The results demonstrate that Tibetan sheep meat subjected to high-temperature and high-pressure processing for controlled durations undergoes a series of chemical reactions, particularly Maillard reactions, leading to enhanced formation of volatile flavor compounds and consequent elevation of ROVA values. Additionally, compared with the APHP and HPAP groups, the HP group exhibited higher ROVA values for compounds with distinctive odors, such as trimethylpyrazine, 2-methyl-1-butanol, and 2-nonanone, indicating that prolonged high-pressure cooking generates more volatile flavor compounds with undesirable odors. Meanwhile, the ROVA values of hexyl acetate and 2-octanone were significantly higher in the AP group than in the other three groups.

### 3.8. GC–MS and GC–IMS Wayne’s Plot Analysis of Volatile Components of Tibetan Sheep Meat After Different Methods of Cooking

**Figure 9:**
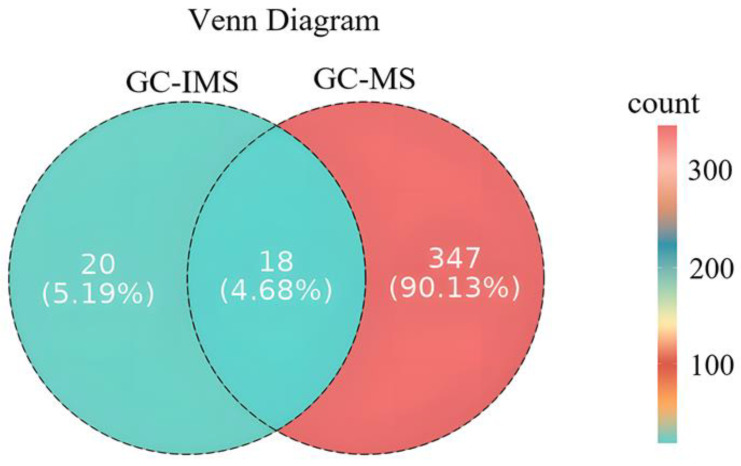
Venn diagram analysis of GC–MS and GC–IMS of Tibetan sheep by different cooking method.

As shown in Figure 9, a total of 403 volatile compounds were detected by GC–IMS and GC–MS in the Tibetan sheep meat after different methods of cooking. Among these, 18 volatiles, including (E)-2-octenal, (E,E)-2,4-heptadienal, (Z)-4-heptenal, benzaldehyde, heptanal, 1-octanol, 1-hexanal, 1-hexanol, 1-octen-3-ol, 1-pentanol, 1-penten-3-ol, 2-heptanone, acetone, ethyl acetate, (E)-2-heptenal, limonene, 2-pentylfuran, and butyric acid, were identified.

### 3.9. Correlation Analysis of Characteristic Flavor Substances with Key Fatty Acids and Amino Acids in Tibetan Sheep Meat After Different Methods of Cooking

**Figure 10:**
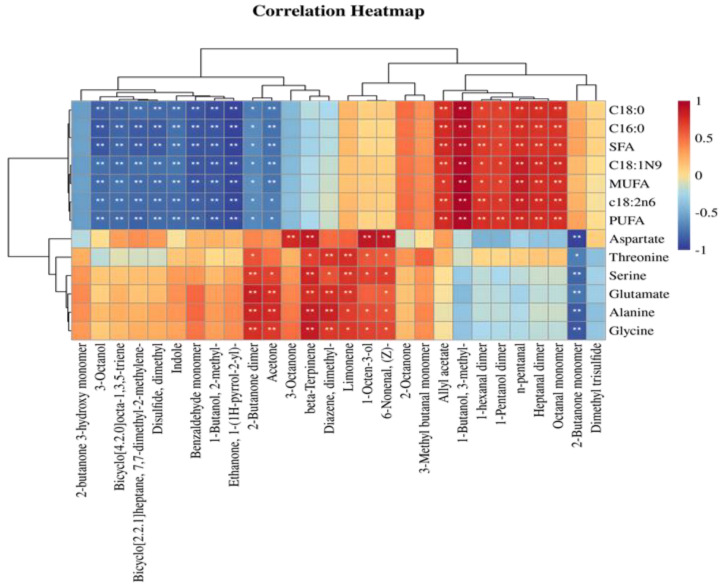
Correlation analysis of characteristic flavor substances and key fatty acids and amino acids of Tibetan sheep by different cooking methods. Note: * indicates that FA and AA are significantly correlated with VOCs (*p* < 0.05); ** indicates that they are highly significantly correlated (*p* < 0.01).

The cooking of Tibetan sheep meat induces chemical reactions that lead to the degradation of fatty acids and amino acids, resulting in the generation of numerous volatile compounds. The relationships between key volatile components and essential fatty acids and amino acids are illustrated in Figure 10. Various types of fatty acids exhibited significant correlations with volatile components during the steaming of Tibetan sheep meat. It was observed that the majority of volatile compounds produced from fatty acid degradation were alcohols, aldehydes, and ketones, aligning with previous research findings [31]. The contents of C18:0, PUFA, C18:1N9, MUFA, C16:0, and SFA were significantly and positively correlated with heptanal dimer, 1-pentanol dimer, octanal monomer, 1-hexanal dimer, n-pentanal, and 3-methyl-1-butanol, indicating that these fatty acids may have facilitated the production of these volatile compounds during the cooking process [45]. Conversely, these fatty acids were significantly negatively correlated with 1-(1H-pyrrol-2-yl) ethanone, benzaldehyde M, indole, 2-methyl-1-butanol, bicyclo[4.2.0]octa-1,3,5-triene, 3-octanol, dimethyldisulfide, and 7,7-dimethyl-2-methylenebicyclo[2.2.1]heptane, suggesting that changes in these fatty acids may affect the cooking process of Tibetan sheep during the formation of aroma components. This study aligns with the recognized significance of lipids in meat aroma production [46]. Previous research has identified C18:1, C18:2, C18:3, and C20:4 as critical substrates in the formation of aroma compounds. Moreover, the accumulation of C18:0 and C18:1N9 may affect aroma retention during the cooking process [47]. Consequently, elucidating the alterations in these fatty acids is crucial for enhancing the flavor profile of Tibetan sheep meat.

Amino acids have been found to have a strong association with volatile components during the steaming of Tibetan sheep meat. Specifically, aspartic acid (Asp) exhibited a positive correlation with 3-octanone, 2-methyl 1-butanol, and β-terpinene, while showing a negative correlation with heptanal dimer, 1-pentanol dimer, octanal M, and 1-hexanal dimer. These findings suggest that Asp may play a role in the production or inhibition of various volatile components through distinct chemical reactions. Concurrently, alanine (Ala), threonine (Thr), glycine (Gly), glutamic acid (Glu), and serine (Ser) exhibited significant positive correlations with acetone, 2-butanone dimer, dimethyl disulfide, β-terpinene, (Z)-6-nonenal, and 1-octen-3-ol, while demonstrating negative correlations with 2-butanone M. These amino acids displayed distinct associations, indicating their potential involvement in the synthesis or inhibition of various volatile compounds through different chemical reactions. The diverse associations of these amino acids suggest their differential roles in the flavor formation of Tibetan sheep. By elucidating the interactions between fatty acids, amino acids, and volatile components, these findings offer a theoretical foundation for the quality control and flavor enhancement of Tibetan sheep, thereby contributing to the advancement of the meat processing industry.

## 4. Conclusions

This study used GC–IMS, GC–MS, and electronic sensory technology to evaluate Tibetan sheep meat, finding that high-pressure steaming is the optimal cooking method. It identified key flavor compounds such as 1-hexanal and (E)-2-octenal, which contribute to fatty, mushroomy, buttery, and caramelized flavors, and high-pressure cooking introduced unique sulfuric and fresh onion odors. Meanwhile, the study on free amino acids and fatty acids revealed that high-pressure steamed Tibetan sheep meat exhibited significantly higher amino acid content but lower fatty acid content, which substantially enhanced the generation of volatile flavor compounds. Furthermore, the research also highlighted strong associations between amino acids, fatty acids, and flavor components, offering insights for future studies on the thermal processing and flavor of Tibetan sheep from Qinghai.

## Figures and Tables

**Table 1:** Cooking conditions of Tibetan sheep.

Cooking Method	Liquid–Solid Ratio	Temperature/°C	Time/h
AP	2:1	100	2
HP	2:1	110	2
APHP	2:1	100/110	1/1
HPAP	2:1	110/100	1/1

Note: AP refers to atmospheric pressure cooking, HP refers to high-pressure cooking, APHP refers to atmospheric pressure high-pressure cooking, and HPAP refers to high-pressure atmospheric pressure cooking.

**Table 2:** Amino acid content and taste activity value (TAV) of Tibetan sheep by different cooking methods.

Types of Amino Acids	Content g/100 g				*p*-Value	Taste Attribute	Taste Threshold g/100 g	TAV			
	AP	APHP	HP	HPAP				AP	APHP	HP	HPAP
Aspartate	21.62 ± 0.33 c	17.99 ± 1.27 b	43.52 ± 2.05 d	14.32 ± 1.41 a	<0.001	Fresh flavor	100	0.22	0.18	0.44	0.14
Glutamate	1124.78 ± 28.45 c	319.32 ± 22.23 a	1220.86 ± 34.07 d	585.45 ± 31.2 b	<0.001	Fresh flavor	30	37.49	10.64	40.70	19.52
Alanine	952.17 ± 5.77 c	455.35 ± 8.05 a	1174.24 ± 12.24 d	810.72 ± 10.15 b	<0.001	Sweet taste	60	15.87	7.59	19.57	13.51
Glycine	2238.11 ± 114.07 c	774.95 ± 20.41 a	2610 ± 58.48 d	1526.69 ± 51.96 b	<0.001	Sweet taste	130	17.22	5.96	20.08	11.74
Serine	880.59 ± 26.01 c	381.26 ± 35.76 a	938.73 ± 35.9 d	583.33 ± 9.47 b	<0.001	Sweet taste	150	5.87	2.54	6.26	3.89
Threonine	1440.63 ± 52.79 c	491.04 ± 10.42 a	1429.44 ± 80.81 c	1038.26 ± 73.95 b	<0.001	Sweet taste	260	5.54	1.89	5.50	3.99
Tyrosine	340.83 ± 18.6 c	147.63 ± 11.4 a	435.32 ± 25.31 d	295.98 ± 6.86 b	<0.001	bitterness	-	-	-	-	-
Histidine	1420.4 ± 374.66 ab	1042.68 ± 126.24 ab	2711.52 ± 1097.02 ab	1535.85 ± 607.79 a	0.065	bitterness	20	71.02	52.13	135.58	76.79
Isoleucine	1270.91 ± 27.66 c	534.07 ± 23.57 a	1468.74 ± 38.37 d	1063.11 ± 25.27 b	<0.001	bitterness	90	14.12	5.93	16.32	11.81
Leucine	1275.26 ± 29.37 b	530.58 ± 26.81 a	1465.11 ± 8.89 b	1335.9 ± 447.98 b	0.004	bitterness	190	6.71	2.79	7.71	7.03
Tryptophan	28.47 ± 0.72 d	13.74 ± 0.53 a	27.18 ± 0.31 c	25.39 ± 0.79 b	<0.001	-	-	-	-	-	-
Phenylalanine	1391.07 ± 72.44 c	496.59 ± 42.26 a	1808.15 ± 19.43 d	1151.2 ± 27.88 b	<0.001	bitterness	90	15.46	5.52	20.09	12.79
Lysine	37.11 ± 7.01 a	43.46 ± 3.19 a	51.97 ± 3.66 b	38.24 ± 2.1 a	0.012	Sweet taste/bitterness	50	0.74	0.87	1.04	0.76
Methionine	383.27 ± 9.54 b	204.79 ± 5.99 a	505.02 ± 10.76 c	398.27 ± 11.65 b	<0.001	Sweet taste/bitterness	30	12.78	6.83	16.83	13.28
Proline	585.88 ± 5.73 c	289.61 ± 8.27 a	698.69 ± 25.23 d	421.99 ± 5.4 b	<0.001	Sweet taste/bitterness	300	1.95	0.97	2.33	1.41
Arginine	3247.85 ± 1007.9 a	2864.69 ± 706.87 a	3885.63 ± 1297.82 a	2694.04 ± 1124.41 a	0.552	Sweet taste/bitterness	50	64.96	57.29	77.71	53.88
Cysteine	0.36 ± 0.01 b	0.27 ± 0 a	0.27 ± 0.01 a	0.28 ± 0 a	<0.001	Sweet taste/bitterness	-	-	-	-	-
Cystine	1431.51 ± 438.73 a	1464.67 ± 313.69 a	1774.43 ± 561.32 a	1631.04 ± 618.89 a	0.821	-	-	-	-	-	-
Valine	1230.38 ± 11.47 c	502.13 ± 4.92 a	1382.71 ± 39.11 d	992.51 ± 10.59 b	<0.001	Sweet taste/bitterness	40	30.76	12.55	34.57	24.81
Essential amino acid	8477.51 ± 585.65 b	3859.08 ± 243.92 a	10,849.84 ± 1298.35 c	7578.73 ± 1208.01 b	<0.001						
Umami amino acids	7584.53 ± 1156.52 b	4432.31 ± 758.84 a	8934.26 ± 1404.65 b	5631.22 ± 1219.13 a	0.003						
Total free amino acids	19,301.2 ± 2231.26 b	10,574.82 ± 1371.88 a	23,631.55 ± 3350.76 c	16,142.57 ± 3067.75 b	<0.001						

Note: Different letters in the same industry indicate significant differences (*p* < 0.05), otherwise the differences are not significant; AP refers to atmospheric pressure cooking, HP refers to high-pressure cooking, APHP refers to atmospheric pressure high-pressure cooking, and HPAP refers to high-pressure atmospheric pressure cooking; TAV refers to taste activity value.

**Table 3:** Fatty acid content of sheep in different cooking methods.

Name	Content/μg/mL				*p*-Value
	AP	HP	APHP	HPAP	
c4:0	0	0	0	0	-
c6:0	0.07 ± 0.00 b	0.06 ± 0.01 a	0.07 ± 0.01 ab	0.08 ± 0.00 c	0.004
c8:0	0.62 ± 0.01 c	0.37 ± 0.01 a	0.52 ± 0.02 b	0.51 ± 0.01 b	<0.001
c10:0	41.78 ± 0.05 d	14.36 ± 0.11 a	31.72 ± 0.05 c	21.45 ± 0.10 b	<0.001
c11:0	1.46 ± 0.02 d	0.5 ± 0.02 a	1.01 ± 0.03 c	0.76 ± 0.01 b	<0.001
c12:0	117.72 ± 0.12 d	41.31 ± 0.14 a	79.68 ± 0.35 c	67.48 ± 0.35 b	<0.001
c13:0	10.08 ± 0.11 d	3.44 ± 0.02 a	7.04 ± 0.05 c	5.27 ± 0.03 b	<0.001
c14:0	1390.07 ± 1.09 d	514.38 ± 1.33 a	974.16 ± 4.23 c	793.88 ± 2.66 b	<0.001
c14:1n5	40.54 ± 0.21 d	16.77 ± 0.19 a	24.73 ± 0.09 c	23.78 ± 0.44 b	<0.001
c15:0	309.3 ± 0.08 d	102.8 ± 0.16 a	219.93 ± 0.95 c	166.08 ± 0.64 b	<0.001
C15:1	0	0	0	0	-
c16:0	7181.61 ± 30.78 d	3461.6 ± 15.48 a	5817.63 ± 17.66 c	4777.99 ± 5.78 b	<0.001
c16:1n7	471.32 ± 1.52 d	228.71 ± 0.31 a	344 ± 0.91 c	316.09 ± 2.86 b	<0.001
c17:0	670.42 ± 1.72 d	240.09 ± 1.19 a	524.59 ± 2.48 c	394.13 ± 0.28 b	<0.001
c17:1n7	193.32 ± 2.37 d	91.99 ± 1.35 a	152.61 ± 1.07 c	134.25 ± 0.56 b	<0.001
c18:0	8100.85 ± 8.69 d	3324.34 ± 14.78 a	6430.79 ± 16.45 c	4683.11 ± 7.27 b	<0.001
c18:1tn9	207.49 ± 2.28 d	74.99 ± 0.16 a	166.8 ± 1.09 c	119.34 ± 1.65 b	<0.001
c18:1n9	9499.59 ± 46.51 d	5382.79 ± 2.17 a	8144.97 ± 13.89 c	6912.5 ± 7.89 b	<0.001
c18:2ttn6	19.19 ± 0.28 c	8.43 ± 0.10 a	19.22 ± 0.18 c	13.47 ± 0.17 b	<0.001
c18:2n6	1254.81 ± 5.40 d	781.65 ± 1.98 a	1095.64 ± 1.74 c	953.46 ± 2.17 b	<0.001
c18:3n6	8.03 ± 0.07 c	5.61 ± 0.03 a	6.77 ± 0.04 b	5.57 ± 0.03 a	<0.001
c18:3n3	604.21 ± 1.56 d	378.65 ± 1.00 a	495.15 ± 1.05 c	421.24 ± 0.68 b	<0.001
c20:0	135.87 ± 0.88 d	41.61 ± 0.29 a	103.33 ± 0.7 c	70.6 ± 0.10 b	<0.001
c20:1n9	28.14 ± 0.15 d	13.42 ± 0.13 a	25.03 ± 0.2 c	24.24 ± 0.17 b	<0.001
c20:2n6	36.39 ± 0.24 c	34.74 ± 0.27 a	37.69 ± 0.16 d	35.91 ± 0.31 b	<0.001
c20:3n6	22.73 ± 0.10 c	19.89 ± 0.22 a	22.12 ± 0.19 b	20.03 ± 0.04 a	<0.001
c20:3n3	8.15 ± 0.02 d	4.84 ± 0.04 a	6.25 ± 0.01 c	5.53 ± 0.07 b	<0.001
c20:4n6	260.19 ± 1.51 b	267.75 ± 2.27 c	274.05 ± 0.66 d	250.24 ± 2.90 a	<0.001
c20:5n3	13.84 ± 0.06 d	3.98 ± 0.06 a	10.11 ± 0.19 c	6.7 ± 0.03 b	<0.001
c21:0	13.65 ± 0.16 d	3.89 ± 0.02 a	9.8 ± 0.07 c	6.56 ± 0.01 b	<0.001
c22:0	272.16 ± 0.62 d	240.61 ± 0.97 c	223.19 ± 2.27 b	212.81 ± 0.86 a	<0.001
c22:1n9	3.65 ± 0.03 b	2.48 ± 0.01 a	4.6 ± 0.02 c	4.85 ± 0.08 d	<0.001
c22:2n6	1.04 ± 0.03 b	0.86 ± 0.02 a	1.96 ± 0.04 d	1.55 ± 0.06 c	<0.001
c22:4n6	9.95 ± 0.1 a	10.49 ± 0.17 b	10.53 ± 0.2 b	11.11 ± 0.1 c	<0.001
c22:5n3	182.94 ± 1.13 d	156.26 ± 0.26 b	162.28 ± 0.77 c	153.64 ± 0.89 a	<0.001
c22:5n6	3.33 ± 0.02 a	4.39 ± 0.04 c	4.81 ± 0.03 d	4.07 ± 0.01 b	<0.001
c22:6n3	5.78 ± 0.03 d	2.1 ± 0.02 a	3.08 ± 0.01 c	2.86 ± 0.05 b	<0.001
c23:0	8.02 ± 0.08 d	1.7 ± 0.01 a	5.04 ± 0.04 c	3.62 ± 0.07 b	<0.001
c24:0	10.44 ± 0.03 d	6.1 ± 0.02 a	8.47 ± 0.09 c	7.13 ± 0.03 b	<0.001
c24:1n9	39.72 ± 0.11 b	45.38 ± 0.3 d	41.61 ± 0.06 c	34.98 ± 0.28 a	<0.001
Total SFA	18,264.12 ± 33.94 d	7997.15 ± 31.78 a	14,436.97 ± 40.20 c	11,211.47 ± 1.81 b	<0.001
Total MUFA	10,483.77 ± 52.57 d	5856.52 ± 2.94 a	8904.35 ± 13.81 c	7570.04 ± 8.79 b	<0.001
Total PUFA	2430.58 ± 6.30 d	1679.63 ± 2.39 a	2149.67 ± 1.09 c	1885.37 ± 2.41 b	<0.001
total_n-3	814.92 ± 2.26 d	545.84 ± 1.03 a	676.87 ± 1.64 c	589.98 ± 1.35 b	<0.001
total_n-6	1615.66 ± 4.56 d	1133.79 ± 1.37 a	1472.80 ± 1.95 c	1295.40 ± 1.25 b	<0.001

Note: Different letters in the same industry indicate significant differences (*p* < 0.05), otherwise the differences are not significant; AP refers to atmospheric pressure cooking, HP refers to high-pressure cooking, APHP refers to atmospheric pressure high-pressure cooking, and HPAP refers to high-pressure atmospheric pressure cooking.

**Table 4:** Key volatile flavor compounds and ROVA values of Tibetan sheep by different cooking methods.

Name	Thresholds mg/kg	ROVA-Value				Odor Characteristics
		AP	HP	APHP	HPAP	
1-Hexanal	0.005	100	100	100	100	Grass, Butter, Fat
(E)-2-Nonenal	0.00008	2.7348	2.4607	4.1682	3.5317	Cucumber, Fat, Green
(Z)-4-Heptenal	0.0034	10.7319	10.8634	11.1558	9.6248	Cookies, Cream
Isovaleraldehyde (CH20)3	0.0016	56.4529	70.3360	42.6913	50.8259	Malt
(E)-2-Octenal	0.003	4.9991	3.7853	7.2082	5.9633	Green, Nuts, Fat
1-Hexanol	0.5	0.2584	0.2129	0.3127	0.4320	Resin, Flower, Green
1-Octanol	0.003	1.2939	1.3530	1.6106	1.86	Green, Nut, Fat
1-Octen-3-ol	0.1	0.9817	0.9918	1.2247	1.3479	Mushroom Flavor
2-Butanone-3-hydroxy	0.055	1.2121	2.0097	0.9106	1.7526	Butter, Cream
2-Butanone	1.3	0.2455	0.2194	0.2611	0.2882	Ether
2-Heptanone	0.140	0.4710	0.3831	0.5839	0.6917	Soap
2-Hexanone	0.098	0.1195	0.1122	0.1409	0.1554	Ether
2-Hexenal	0.030	1.6359	0.9550	1.8395	1.5493	Fat, Rancid
2-Methylpropyl butyrate	0.0094	0.8500	1.0393	1.1576	1.4023	-
2-Pentylfuran	0.006	4.6876	4.1328	6.2467	7.8197	Green beans, Butter
2-Phenylacetaldehyde	0.004	2.0281	1.8077	3.3242	3.1988	Hawthorn, Honey, Sweet
Benzaldehyde	0.350	0.4207	0.7625	0.4772	0.5414	Almond, Caramel
butanal	0.009	3.8087	4.0925	2.8226	3.3521	Pungent, Green
Butyl 2-methylbutyrate	0.017	0.2022	0.3982	0.5332	0.4441	Fruit, Cocoa Nibs
Ethyl hexanoate	0.001	8.6336	12.0343	9.0295	12.0622	Apple Peel, Fruit
Heptanol	0.023	1.0280	0.8286	1.3581	1.4492	Citrus
Limonene	0.01	1.3925	2.7656	1.6886	3.8007	Lemon, Orange
Pentanal	0.012	11.2752	10.3673	10.6086	10.0669	Almond, Malt, Spicy

Note: AP refers to atmospheric pressure cooking, HP refers to high-pressure cooking, APHP refers to atmospheric pressure high-pressure cooking, and HPAP refers to high-pressure atmospheric pressure cooking; ROVA refers to relative odor value activity

**Table 5:** Key volatiles and ROVA values (GC–MS) of Tibetan sheep by different cooking method.

Name	Thresholds (mg/kg)	ROVA-Value				Odor Characteristics
		AP	APHP	HP	HPAP	
3-Methyl-1-butanol	0.2500	100.00	100.00	100.000	100.00	Whisky, malt, caramelized flavors
Methyl butyrate	0.0151	5.03	0.075	0.30	0.08	Fruity, sweet
2-Ethylpyridine	0.0570	3.02	2.771	13.88	2.82	Grassy
Vinyl propionate	0.0400	3.16	0.220	39.04	0.69	-
3-(methylthio)-1-propanol	0.1230	4.29	1.659	1.50	1.29	Sweet, departmental
S-methyl propylthioate	0.0500	10.00	58.312	36.96	27.71	-
Trimethylpyrazine	0.0230	9.13	23.553	83.09	24.13	Barbecue, potato
2-methyl-1-butanol	0.3000	0.01	4.054	85.00	8.48	Wine, onion flavor
2-Octanol	0.0500	4.08	11.778	46.92	11.68	Mushroom, fat.
p-Xylene	0.6000	1.67	1.216	5.82	0.28	-
2-Pentanol	1.0000	1.35	3.726	9.11	0.77	Green
2-Nonanone	0.2000	19.34	24.355	28.60	10.35	Hot milk, soap, green
Dimethyl disulfide	0.0120	1.71	18.94	58.69	18.54	Onion, cabbage, putrid odor
3-Methylphenol	0.0004	3.06	5.63	16.59	8.90	Feces, plastic
3-Methyl-2-hexanone	0.0410	11.57	4.929	4.77	2.26	-
2-Ethyl-1-hexanol	0.3000	3.98	1.642	13.98	1.14	Rose odor, green
5-Pentyl dihydro-2(3H)-furanone	0.0097	12.04	19.792	82.79	29.38	Coconut, peach flavor
5-Ethyldihydro-2(3H)-furanone	0.0500	1.93	6.935	15.01	2.70	Coumarin, sweet
(E,E)-2,4-heptadienal	0.0154	12.58	8.076	32.67	8.67	Nutty, fatty
4-ethylbenzaldehyde	0.0130	13.28	1.777	40.01	4.40	Sweet
(1R)-2,6,6-trimethylbicycl0[3.1.1] hept-2-ene	0.0046	11.23	13.677	57.03	7.95	-
hexyl acetate	0.0020	52.63	5.020	13.19	0.76	Fruity, herbal
(-)-Carvone	0.0020	2.76	23.292	76.38	67.41	Mint flavor
1-Hexanol	0.5000	13.62	18.696	30.04	28.80	Resinous, floral, green
(E,E)-3,5-octadien-2-one	0.1000	3.66	3.176	11.85	3.47	-
1-Octen-3-ol	0.0010	11.26	69.160	47.43	27.94	Mushroom flavor
2-Octanone	0.0500	20.06	5.047	8.42	6.52	Soap, gasoline
1,3-Dimethylbenzene	1.1000	1.53	1.516	5.17	0.99	Plastic
2-methyl-1-pentanol	0.8300	3.36	4.595	11.30	2.44	Green, seaweed, cucumber
(E,E)-2,4-octadienal	0.0100	12.92	10.829	37.00	12.38	Green, seaweed, cucumber
3-Pentanone	0.0400	75.97	25.299	89.14	12.68	Ether
β-Terpinene	0.2600	10.26	2.460	94.46	6.01	Gasoline, turpentine
hexanal	0.0050	2.30	3.318	5.66	1.59	Grass, tallow, fat
(Z)-2-decenal	0.0022	11.60	10.886	88.16	7.68	Tallow

Note: AP refers to atmospheric pressure cooking, HP refers to high-pressure cooking, APHP refers to atmospheric pressure high-pressure cooking, and HPAP refers to high-pressure atmospheric pressure cooking; ROVA refers to relative odor value activity.

## Data Availability

The original contributions presented in this study are included in the article. Further inquiries can be directed to the corresponding author.

## Supplementary Materials

The following supporting information can be downloaded at: https://www.mdpi.com/article/10.3390/foods14132181/s1, Figure S1. Electronic nose results map of saline-alkali ground Tibetan sheep in different cooking methods; Figure S2. Analysis of electronic tongue principal components of volatile sheep in different cooking methods; Table S1. Feed composition table; Table S2. Sensory evaluation form; Table S3. Sensory evaluation data of Tibetan sheep with different cooking methods; Table S4. Volatile components of Tibetan sheep by different cooking methods; Table S5. Volatile ingredients of Tibetan sheep (GC-IMS, *P* < 0.05, VIP > 1); Table S6. Analysis of Tibetan sheep volatile substances in different cooking methods (GC-MS); Table S7. Volatile ingredients of Tibetan sheep (GC-MS, VIP > 1.5, *P* < 0.05).

## Abbreviations

The following abbreviations are used in this manuscript:

AP	Atmospheric Pressure
HP	High-Pressure
APHP	Atmospheric-High Pressure
HPAP	High-Atmospheric Pressure
GC–IMS	Gas Chromatography–Ion Mobility Spectrometry
GC–MS	Gas Chromatography–Mass Spectrometry
ROVA	Relative Oder Activity Value
VIP	Variable Influence on Projection
SFA	Saturated Fatty Acid
PUFA	Polyunsaturated Fatty Acid
MUFA	Monounsaturated Fatty Acid
LDL	Low-Density Lipoprotein
TAV	Taste Activity Value
OPLS-DA	Orthogonal Partial Least Squares-Discriminant Analysis
ASTREE	Abstract Syntax Tree
