# Supplementary material for: Influence of Cooking Methods on Flavor Parameters and Sensory Quality of Tibetan Sheep Meat Examined Using an Electronic Nose, an Electronic Tongue, GC–IMS, and GC–MS
Source: Foods. 2025 Jun 22;14(13):2181. doi: 10.3390/foods14132181 (PMC12248567; doi:10.3390/foods14132181)
Supplement: Supplementary file 1 [file foods-14-02181-s001.zip › foods-3674200-supplementary.pdf]

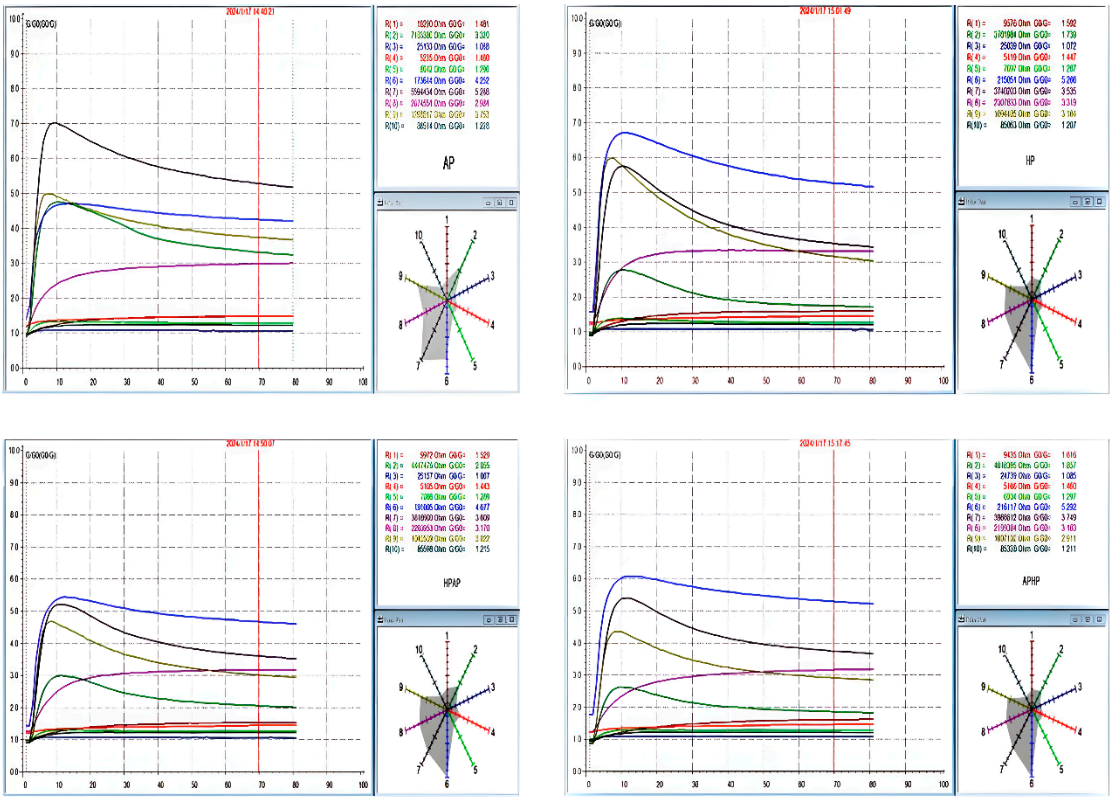

Figure S1. Electronic nose results map of saline-alkali ground Tibetan sheep in different cooking methods

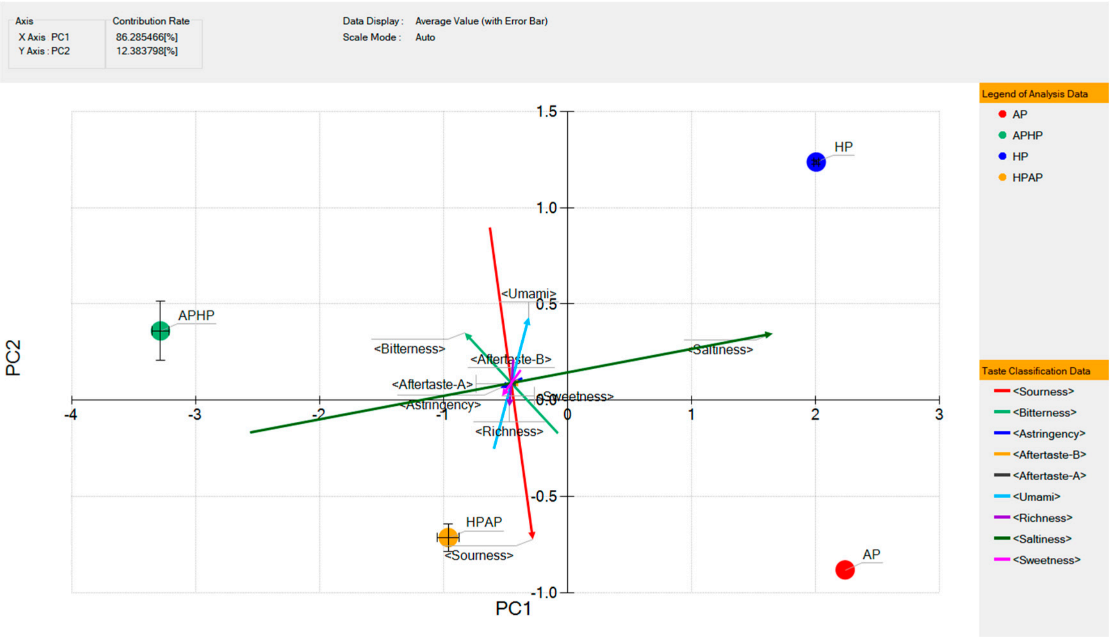

Figure S2. Analysis of electronic tongue principal components of volatile sheep in different cooking methods

Figure S1. Electronic nose results map of saline-alkali ground Tibetan sheep in different cooking methods

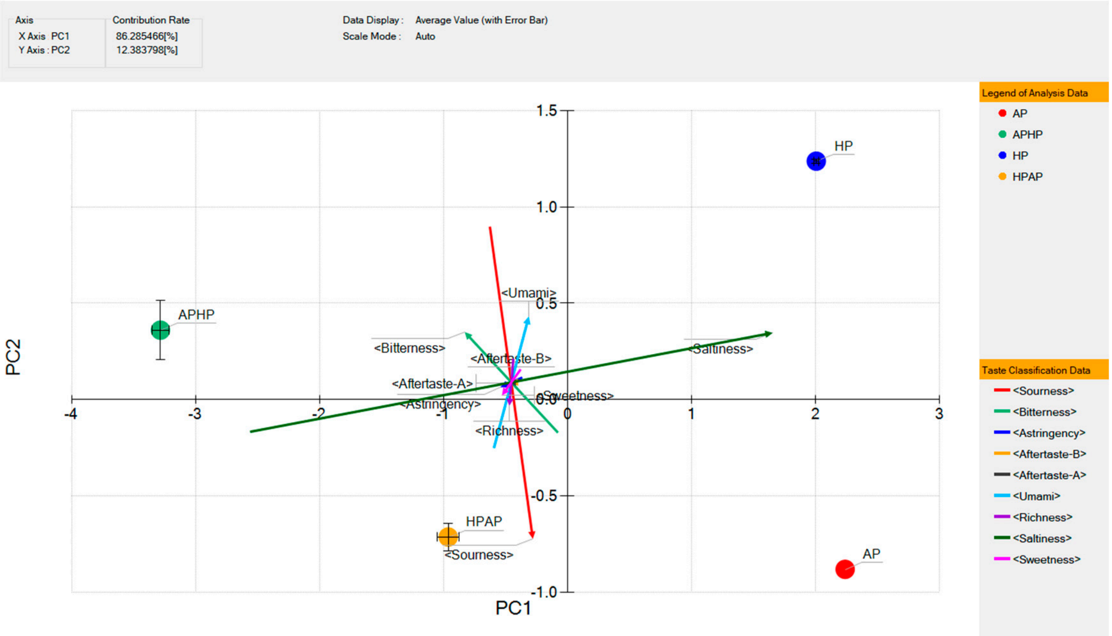

Table S1. Feed composition table

Items	Content
Forage（30%）	
Oat green hay	50
Oat silage	50
Total	100
Balanced concentrate（70%）	
Corn	52
Wheat	9
Palm kernel meal	16
Soybean meal	4
Rapeseed meal	15
NaCl	1
Limestone	1
Baking soda	1
1%Premix	0.6
4% Concentrate feed	0.4
Total	100

Table S2. Sensory evaluation form

**Evaluate the project**		**Score/Description**		
		**1-4**	**5-7**	**8-10**
Appearance	Color and lustre	Vibrant colors	The color is relatively dim	The color is dark

**Evaluate the project**		**Score/Description**		
		**1-4**	**5-7**	**8-10**
Smell	Aroma	No meaty aroma	The meat has a strong aroma	Meat aroma is abundant
	Gamy odor	Obvious gamey smell	No obvious gamey smell	No gamey smell
Texture		Rough meat texture and poor chewiness	The meat is fresh, tender, and delicious, with average chewiness	Fresh and tender, with good chewiness
Texture state		Poor organizational status, hard and aged meat	The organizational status is average, and the elasticity is average	Uniform and tight organizational state, good flexibility, and uniform internal structure
Overall acceptability		Not easily accepted	More easily accepted	Easy to accept

Table S3. Sensory evaluation data of Tibetan sheep with different cooking methods

	**AP**	**HP**	**APHP**	**HPAP**
Color and lustre	$7.83{\pm}0.75^{a}$	$7.33{\pm}0.52^{a}$	$7.00{\pm}0.89^{a}$	$7.00{\pm}0.63^{a}$
Smell	$7.17{\pm}0.75^{a}$	$8.50{\pm}0.55b^{c}$	$8.83{\pm}0.75^{b}$	$8.00{\pm}0.00^{c}$
Taste	$8.17{\pm}0.41^{b}$	$7.00{\pm}1.10^{a}$	$8.33{\pm}0.82^{b}$	$8.33{\pm}0.52^{b}$
Texture	$6.33{\pm}0.52^{a}$	$8.67{\pm}0.52^{b}$	$8.00{\pm}0.89^{b}$	$7.00{\pm}0.63^{a}$
Acceptable level	$7.67{\pm}0.82^{a}$	$8.33{\pm}0.52^{ab}$	$8.67{\pm}0.52^{b}$	$8.00{\pm}0.63^{ab}$

Note: There is a significant difference (P<0.05) between different letters, otherwise the difference is not significant. AP stands for atmospheric pressure cooking, HP stands for high-pressure cooking, APHP stands for atmospheric pressure high-pressure cooking, HPAP stands for high-pressure atmospheric pressure cooking.

Table S4. Volatile components of Tibetan sheep by different cooking methods

Type	Compound name	CAS	Retention index	Retention time/s	Migration time/ms	Peak volume				*P*-value	VIP value	Odor characteristic
						AP	HP	APHP	HPAP			
Aldehydes (27)	(E)-2-Nonenal	C18829566	1151.70	896.54	1.41	234.46±12.61^{b}	149.84±22.34^{a}	253.13±1.74^{b}	234.04±30.59^{b}	<0.01	1.15	Cucumber,Fat,Green
	(E)-2-Octenal D	C2548870	1068.60	708.12	1.82	39.26±1.90^{ab}	33.00±4.42^{a}	46.68±2.97^{b}	37.70±8.75^{ab}	0.68	2.71	Green, Nuts, Fat
	(E)-2-Octenal M	C2548870	1067.20	705.47	1.34	284.36±23.92^{b}	143.73±6.15^{a}	354.69±26.79^{c}	316.57±17.33^{b}	<0.01	0.91	Green, Nuts, Fat
	(E,E)-2,4-heptadienal	C4313035	1024.60	625.21	1.19	120.45±10.16^{b}	75.91±4.49^{a}	166.33±18.45^{bc}	141.98±16.6^{b}	<0.01	1.49	Nuts, Fat
	(Z)-4-heptenal	C6728310	901.10	396.79	1.15	787.36±15.21^{d}	574.85±11.61^{a}	704.00±24.19^{c}	648.04±10.59^{b}	<0.01	0.30	Cookies, Cream
	(E)-2-heptenal D	C18829555	960.90	501.99	1.67	173.16±15.32^{a}	131.54±1.70^{a}	273.37±46.70^{b}	253.72±23.87^{b}	<0.01	1.53	Soap, Fat, Almonds
	(E)-2-heptenal M	C18829555	960.40	500.97	1.26	594±33.86^{b}	366.33±26.97^{a}	732.36±41.73^{c}	655.48±20.74^{d}	<0.01	0.69	Soap, Fat, Almonds
	(E)-2-Pentenal D	C1576870	753.50	232.35	1.36	258.54±9.89^{b}	61.43±5.43^{a}	308.97±44.17^{c}	223.38±24.38^{b}	<0.01	0.86	Strawberry,Fruit,Tomato
	(E)-2-pentenal M	C1576870	753.50	232.35	1.11	546.02±7.87^{c}	302.86±14.50^{a}	553.52±31.94^{c}	491.82±21.52^{b}	<0.01	0.56	Strawberry,Fruit,Tomato
	n-Nonenal D	C124196	1104.70	784.54	1.94	661.59±40.13^{b}	464.74±26.81^{a}	620.50±79.06^{b}	928.06±71.28^{c}	<0.01	1.20	Fat, Citrus, Green

Type	Compound name	CAS	Retention index	Retention time/s	Migration time/ms	Peak volume				*P*-value	VIP value	Odor characteristic
						AP	HP	APHP	HPAP			
	n-Nonenal	C124196	1104.20	783.40	1.48	1889.08±161.06^{b}	2069.48±124.01^{a}	2373.11±195.29^{b}	2964.08±76.17^{c}	<0.01	1.32	Fat, Citrus, Green
	n-Pentanal	C110623	701.00	189.98	1.42	2919.61±60.03^{c}	1936.23±139.73^{a}	2362.84±108.4^{b}	2392.23±8.25^{b}	<0.01	0.08	Almonds,Malt, Spicy
	Octanal D	C124130	1013.20	605.22	1.82	2845.67±62.92^{c}	1365.24±92.44^{a}	2323.03±359.47^{b}	3233.38±144.74^{d}	<0.01	0.86	Fat, Soap, Lemon, Green
	Octanal M	C124130	1012.80	604.58	1.40	3141.92±73.14^{d}	2385.41±40.35^{a}	2756.11±95.04^{b}	2935.11±21.64^{c}	<0.01	0.20	Fat, Soap, Lemon, Green
	2-Hexenal D	C505577	853.10	333.68	1.52	279.53±15.79^{b}	78.05±9.65^{b}	284.85±44.63^{b}	254.76±21.95^{b}	<0.01	1.06	Fat, Rancid
	2-Hexenal M	C505577	852.10	332.45	1.18	779.5±22.17c	367.83±12.62^{a}	739.41±47.27^{c}	665.63±21.23^{b}	<0.01	0.34	Fat, Rancid
	2-Phenylacetaldehyde	C122781	1043.90	660.31	1.27	175.05±6.81^{b}	112.54±2.14^{a}	246.79±23.41^{c}	253.38±15.19^{c}	<0.01	0.89	Hawthorn, Honey, Sweet
	Isovaleraldehyde D	C590863	659.30	168.22	1.41	349.43±33.78^{c}	455.82±62.54^{d}	130.83±21.56^{a}	263.34±13.82^{b}	<0.01	0.48	Malt
	Isovaleraldehyde M	C590863	659.80	168.47	1.19	1599.64±49.55^{c}	1295.68±28.61^{b}	1136.98±71.15^{a}	1347.06±23.01^{b}	<0.01	0.36	Malt
	Benzaldehyde	C100527	964.90	509.8	1.15	1263.84±77.5	2051.73±98.2^{c}	1322.19±114.57^{a}	1779.16±47.59^{b}	<0.01	0.41	Almond,

Type	Compound name	CAS	Retention index	Retention time/s	Migration time/ms	Peak volume				P-value	VIP value	Odor characteristic
						AP	HP	APHP	HPAP			
	M			3		5[a]						Caramel
	Benzaldehyde D	C100527	965.10	510.31	1.47	1913.18±112.46[b]	2101.66±63.56[c]	1777.98±32.75[a]	1973.09±16.79[b]	0.02	0.33	Almond, Caramel
	n-Butyraldehyde	C123728	602.90	145.90	1.29	739.67±11.80[c]	573.24±13.38[b]	471.51±39.4[a]	597.43±10.89[b]	<0.01	0.42	Pungent, Green
	Heptaldehyde D	C111717	904.40	401.98	1.69	5254.91±124.65[d]	3135.32±34.46[a]	4399.15±275.87[b]	4789.77±111.41[c]	<0.01	0.40	Fat, Citrus
	Heptaldehyde M	C111717	902.40	398.90	1.34	2484.16±106.89[b]	2250.48±18.79[a]	2304.67±16.18[a]	2295.65±4.46[a]	0.04	0.86	Fat, Citrus
	1-Hexanal D	C66251	795.90	272.52	1.56	8763.83±353.71[d]	6022.08±78.19[a]	7433.86±280.83[b]	8115.23±297.19[c]	<0.01	1.35	Grass, Butter, Fat
	1-Hexanal M	C66251	794.90	271.50	1.26	2025.41±182.76[b]	1759.72±28.13[a]	1846.49±21.51[a]	1786.18±34.00[a]	0.34	1.82	Grass, Butter, Fat
	2-Furaldehyde	C98011	833.60	311.46	1.09	37.55±1.41[a]	77.74±2.45[b]	84.43±2.59[b]	41.69±7.43[a]	<0.01	0.40	Bread,Almond,Sweet
Alcohols(11)	1-Hexanol D	C111273	873.40	358.55	1.64	524.39±16.2[b]	226.76±23.60[a]	554.61±129.24[b]	1177.24±126.92[c]	<0.01	0.83	Resin, Flower, Green
	1-Hexanol M	C111273	874.10	359.46	1.33	2263.78±86.15[b]	1429.74±61.67[a]	2347.01±238.62[b]	3100.6±104.31[c]	<0.01	0.79	Resin, Flower, Green
	1-Hexanol	C111273	873.20	358.2	1.99	34.59±4.53[a]	28.53±2.51[a]	38.92±5.97[a]	74.52±17.07[b]	0.01	1.79	Resin,

Type	Compound name	CAS	Retention index	Retention time/s	Migration time/ms	Peak volume				*P*-value	VIP value	Odor characteristic
						AP	HP	APHP	HPAP			
	polymer			5								Flower, Green
	1-Octen-3-ol D	C3391864	985.20	552.10	1.60	193±24.73^{b}	142.91±13.25^{a}	275.75±31.42^{c}	374.5±29.79^{d}	<0.01	1.15	Mushroom
	1-Octen-3-ol M	C3391864	984.80	551.33	1.16	1925.26±4.63^{b}	1400.7±28.18^{a}	1997.45±139.46^{b}	2294.73±49.66^{d}	<0.01	0.68	Mushroom
	1-Pentanol D	C71410	763.70	241.68	1.51	2092.68±130.63^{c}	853.81±32.4^{a}	1672.08±148.8^{b}	1988.83±57.87^{c}	<0.01	0.20	Balsamic, balsamic
	1-Penten-3-ol M	C71410	764.40	242.36	1.26	2212.95±60.73^{c}	1751.91±23.21^{a}	2100.19±34.03^{b}	2113.42±4.49^{b}	<0.01	0.10	Balsamic, Balsamic Oil
	1-Penten-3-ol	C616251	689.20	181.56	0.94	598.99±16.63^{c}	307.36±19.26^{a}	454.31±23.68^{b}	437.45±1.44^{b}	<0.01	0.10	Butter, Pungent
	Heptanol D	C5353533	980.50	542.04	1.76	36.05±3.55^{b}	23.23±5.19^{ab}	35.13±6.48^{a}	44.6±8.85^{b}	0.21	2.33	Citrus
	Heptanol M	C53535334	980.50	542.04	1.40	474.17±35.75^{bc}	273.37±19.25^{a}	544.62±74.11^{b}	615.48±34.91^{c}	<0.01	1.20	Citrus
	1-Octanol	C2548870	1081.30	734.10	1.47	83.76±5.82^{b}	63.17±7.74^{a}	89.68±13.58^{b}	113.98±12.11^{c}	0.02	1.96	Green, Nutty, Fatty
Ketones(10)	2-Butanone-3-hydroxyD	C513860	712.20	198.30	1.33	355.12±32.05^{b}	419.16±31.78^{c}	223.65±19.64^{a}	504.22±31.06^{d}	<0.01	0.49	Butter, Creamy
	2-Butanone-3-hydroxyM	C513860	714.40	200.00	1.06	1083.47±80.01^{b}	1301.1±66.51^{c}	705.94±23.36^{a}	1404.62±12.89^{d}	<0.01	0.36	Butter, Cream

Type	Compound name	CAS	Retention index	Retention time/s	Migration time/ms	Peak volume				*P*-value	VIP value	Odor characteristic
						AP	HP	APHP	HPAP			
	2-Butanone D	C78933	588.50	140.69	1.24	5490.78±87.76^{b}	6222.6±79.31^{c}	4461.11±260.98^{a}	5568.37±123.02^{b}	<0.01	0.10	Ether
	2-Butanone M	C78933	588.50	140.69	1.07	1394.68±166.56^{b}	1048.6±46.42^{a}	1839.8±36.8^{c}	1849.9±16.55^{c}	<0.01	0.41	Ether
	2-Heptanone D	C110430	892.20	383.27	1.63	442.12±33.89^{b}	203.07±11.21^{a}	529.06±93.98^{b}	788.51±57.17^{c}	<0.01	1.01	Soap
	2-Heptanone M	C110430	891.50	382.27	1.26	980.66±10.06^{b}	631.61±19.72^{a}	988.3±76.41^{b}	1129.14±19.15^{c}	<0.01	0.55	Soap
	2-Hexanone D	C591786	782.40	259.61	1.50	97.95±3.44^{b}	29.65±3.18^{a}	77.69±19.48^{b}	100.94±8.48^{bc}	<0.01	0.98	Ether
	2-Hexanone M	C591786	783.60	260.84	1.19	154.71±1.71^{a}	141.45±3.8^{a}	178.65±17.22^{b}	200.73±8.41^{c}	<0.01	1.35	Ethyl ether
	Acetone	C67641	501.20	112.86	1.13	1943.60±100.80^{b}	2878.32±165.4^{d}	1162.52±109.55^{a}	2243.14±54.93^{c}	<0.01	0.19	Pungent
	3-Methyl-2(5H)-furanone	C22122367	980.50	542.04	1.11	87.91±9.11^{b}	70.18±4.55^{a}	75.54±3.48^{a}	92.71±7.47^{b}	0.10	1.92	-
	2-Methylpropyl butyrate	C539902	958.00	496.18	1.33	172.41±11.10^{a}	152.04±2.64^{a}	201.96±20.99^{b}	261.04±7.73^{c}	<0.01	1.01	-
Esters(6)	Ethyl acetate D	C141786	611.20	148.98	1.34	143.18±4.76^{c}	84.53±4.28^{b}	62.86±9.23^{a}	90.32±1.15^{b}	<0.01	0.26	Pineapple
	Ethyl acetate M	C141786	611.20	148.98	1.11	436.26±19.7^{9}c	328.69±3.05^{a}	351.35±18.3^{a}	379.44±4.35^{b}	<0.01	0.04	Pineapple

Type	Compound name	CAS	Retention index	Retention time/s	Migration time/ms	Peak volume				*P*-value	VIP value	Odor characteristic
						AP	HP	APHP	HPAP			
	Butyl 2-methylbutyrate	C15706737	1043.50	659.61	1.37	74.19±9.90^{a}	105.35±11.68^{b}	168.23±9.23^{d}	149.51±4.54^{c}	<0.01	0.36	Fruit, Cocoa Nibs
	Ethyl caproate	C123660	999.10	581.57	1.33	186.3±2.74^{a}	187.3±8.60^{a}	167.59±16.02^{a}	238.87±11.69^{c}	<0.01	1.34	Apple Peel, Fruit
	Gamma-butyrolactone	C96480	924.60	435.25	1.08	61.23±1.11^{b}	80.51±10.01^{c}	43.46±3.33^{a}	61.55±4.70^{b}	<0.01	0.82	Caramel, Sweet
Olefins（2）	Limonene D	C138863	1040.80	654.54	1.30	159.59±0.9a	230.81±11.65^{b}	168.86±5.36^{a}	402.44±4.48^{c}		0.11	Lemon, Orange
	Limonene M	C138863	1040.80	654.54	1.22	140.90±4.50^{a}	199.61±14.74^{b}	144.55±8.99^{a}	350.20±5.57^{c}		0.16	Lemon, Orange
Heterocycles(1)	2-Pentylfuran	C3777693	996.70	577.62	1.25	606.91±42.96^{b}	385.93±29.69^{a}	695.66±80.12^{b}	929.11±52.80^{c}	<0.01	0.93	Green Bean, Butter
Acids（1）	Butyric acid	C107926	816.00	292.63	1.16	150.79±3.06^{c}	102.75±11.19^{a}	120.97±10.24^{b}	155.07±5.84^{c}	<0.01	1.05	Rancid,Cheese,Sweat

Note: D: dimer, M: monomer, different letters in the same row indicate significant differences ($P<0.05$) and vice versa. Odor characteristics were obtained from the odor description database flavornet and human odor space. Below; AP refers to atmospheric pressure cooking, HP refers to high-pressure cooking, APHP refers to atmospheric pressure high-pressure cooking, HPAP refers to high-pressure atmospheric pressure cooking; CAS refers to chemical abstracts service; VIP refers to variable influence on projection.

Table S5. Volatile ingredients of Tibetan sheep (GC-IMS, *P<0.05*, VIP> 1)

Compound name	Quantity contained(mg/kg)				*P-value*	VIP	Odor Characteristics
	AP	APHP	HP	HPAP			
1-hexanal dimer	8763.83±353.71[d]	6022.08±78.19[a]	7433.86±280.83[b]	8115.23±297.19[c]	<0.01	2.21	Grass, Butter, Fat
2-Butanone dimer	5490.78±87.76[b]	6222.60±79.31[c]	4461.11±260.98[a]	5568.37±123.02b	<0.01	2.01	Ether
Acetone	1943.6±100.80[b]	2878.32±165.40[d]	1162.52±109.55[a]	2243.14±54.93[c]	<0.01	1.93	Pungent
Heptanal dimer	5254.91±124.65[d]	3135.32±34.46[a]	4399.15±275.87[b]	4789.77±111.41[c]	<0.01	1.89	Fat, Citrus
Octanal dimer	2845.67±62.92[c]	1365.24±92.44[a]	2323.03±359.47[b]	3233.38±144.74[d]	<0.01	1.77	Fat, Soap, Lemon, Green
1-Hexanol monomer	2263.78±86.15[b]	1429.74±61.67[a]	2347.01±238.62[b]	3100.6±104.31[c]	<0.01	1.70	Resin, Flower, Green
1-Pentanol dimer	2092.68±130.63[c]	853.81±32.40[a]	1672.08±148.80[b]	1988.83±57.87[c]	<0.01	1.45	Balsamic, Fragrance Oil
1-Hexanol dimer	524.39±16.20[b]	226.76±23.60[a]	554.61±129.24[b]	1177.24±126.92[c]	<0.01	1.40	Resin, Floral, Green
n-pentanal	2919.61±60.03[c]	1936.23±139.73[a]	2362.84±108.4[b]	2392.23±8.25[b]	<0.01	1.39	Almond, Malt, Spicy
2-butanone3-hydroxy monomer	1083.47±80.01[b]	1301.1±66.51[c]	705.94±23.36[a]	1404.62±12.89[d]	<0.01	1.37	Butter, Cream
Benzaldehyde monomer	1263.84±77.55[a]	2051.73±98.2[c]	1322.19±114.57[a]	1779.16±47.59[b]	<0.01	1.36	Almond, Caramel
n-Nonanal monomer	2368.27±161.06[b]	2069.48±124.01[a]	2373.11±195.29[b]	2964.08±76.17[c]	<0.01	1.29	Fatty, Citrus, Green
2-Butanone monomer	1394.68±166.56[b]	1048.6±46.42[a]	1839.8±36.8[c]	1849.9±16.55[c]	<0.01	1.22	Ether
1-Octen-3-ol monomer	1925.26±4.63[b]	1400.7±28.18[a]	1997.45±139.46[b]	2294.73±49.66[d]	<0.01	1.17	Mushroom
Octanal monomer	3141.92±73.14[d]	2385.41±40.35[a]	2756.11±95.04[b]	2935.11±21.64[c]	<0.01	1.15	Fat, Soap, Lemon, Green
3-Methylbutanal monomer	1599.64±49.55[c]	1295.68±28.61[b]	1136.98±71.15[a]	1347.06±23.0[1b]	<0.01	1.05	Malt

Compound name	Quantity contained(mg/kg)				*P-value*	VIP	Odor Characteristics
	AP	APHP	HP	HPAP			
2-Heptanone dimer	442.12±33.89[b]	203.07±11.21[a]	529.06±93.98[b]	788.51±57.17[c]	<0.01	1.01	Soap

Note: HP refers to high-pressure cooking, APHP refers to atmospheric pressure high-pressure cooking, HPAP refers to high-pressure atmospheric pressure cooking; VIP refers to variable influence on projection.

Table S6. Analysis of Tibetan sheep volatile substances in different cooking methods (GC-MS)

Compound name	CAS	AP	APHP	HP	HPAP	*P*-vaule
Aldehydes（47 species）						
2,4,5-Trimethylbenzaldehyde	5779-72-6	393.44±11.45	421.57±7.89	414.09±9.00	400.16±20.02	0.10
1-Hexenal	505-57-7	2139.47±1125.80	131.19±66.95	244±111.26	106.04±99.21	0.01
2-2-Dimethylhexanal	996-12-3	36.97±24.63	2.24±1.16	12.28±5.34	3.60±3.03	0.03
(E,Z)-2,6,-Renadienal	557-48-2	197.51±98.06	90.31±5.32	1850.34±1684.89	101.79±41.06	0.09
cis-9-hexadecenal	56219-04-6	20866.07±11042.73	20517.91±14764.51	37593.69±5851.16	16065.25±9529.35	0.15
hexadecanal	629-80-1	0.31±0.03	2.04±2.08	0.33±0.19	5.29±1.46	0.00
(Z)-13-octadecadienal	58594-45-9	22.28±20.89	13.76±4.25	12.71±3.10	17.13±7.51	0.74
4-pentylbenzaldehyde	6853-57-2	2220.89±109.91	1304.38±167.03	1466.42±187.08	1406.3±176.77	<0.01
cis-5-dodecenal	68820-33-7	2.72±1.83	1.42±0.44	4.01±2.75	7.29±5.44	0.21
2,6-dodecadien-1-aldehyde	21662-13-5	4.94±2.79	698.32±294.94	380±255.02	694.42±605.93	0.13
(E)-2-butenal	123-73-9	2.04±0.78	6.43±5.70	14.26±23.30	0.24±0.42	0.52
5-Ethylcyclopent-1-enecarboxaldehyde	36431-60-4	2352.91±410.76	2063.08±658.6	2545.4±2085.86	2205.7±257.64	0.96
Pentadecanal	2765-11-9	929.29±474.03	519.29±411.39	51.12±10.18	19.27±14.95	0.02
2-Ethyl-2-hexenal	645-62-5	47214.67±11094.25	35440.15±4496.66	24035.16±1134.34	47122.58±5230.81	0.01
Tetradecanal	124-25-4	333.27±28.71	229.25±38.46	308.29±18.42	186.44±12.37	<0.01
Butyraldehyde C	53447-48-6	260.52±34.53	19.92±13.50	52.90±42.58	301.29±63.32	<0.01
Phenylacetaldehyde	122-78-1	2636.38±199.98	3852.91±693.19	4606.74±336.9	1684.6±314.10	<0.01
Benzaldehyde	100-52-7	16176.41±3999.59	5202.05±1242.31	12527.91±5573.78	3880.61±1289.74	0.01
(E,E)-2,4-Hexadienal	142-83-6	19.9±9.74	1.77±0.12	69.43±40.05	13.37±5.04	0.02
Benzaldehyde dimethyl acetal	1125-88-8	1293.53±829.71	237.49±51.07	1071.79±773.76	158.84±158.14	0.09
Heptanal	111-71-7	19796.20±17539.60	13730.49±1677.76	22250.66±10296.56	7958.11±3787.63	0.39

Compound name	CAS	AP	APHP	HP	HPAP	*P*-vaule
13-Methyltetradecanal	75853-51-9	21.34±19.06	6.21±3.34	13.06±10.07	25.96±20.89	0.44
Dodecanal	112-54-9	1.74±0.55	1202.93±447.50	2612.73±1592.20	364.76±331.21	0.02
undecanal	112-44-7	31012.89±8580.80	90686.38±45733.10	123845.32±79307.11	110210.76±37631.85	0.19
(E)-2-Octenal	2548-87-0	12793.07±3046.74	5670.06±1142.85	5817.32±130.99	4199.83±3202.71	0.01
(E)-2-Dodecenal	20407-84-5	225.78±130.31	534.73±519.79	357.08±237.26	207.21±81.81	0.54
4-Ethylbenzaldehyde	4748-78-1	4902.71±1185.74	472.39±354.80	3270.97±2609.63	754.15±567.16	0.02
2-Undecenal	2463-77-6	5992.25±2172.64	1820.89±1090.62	4752.66±1079.34	1646.24±837.79	0.01
Renal	124-19-6	30756.5±7859.85	18179.69±6249.25	35423.3±12344.65	27529.95±6180.25	0.17
(E,Z)-2,4-decadienal	25152-83-4	1078.21±505.31	248.67±174.26	779.96±185.61	298.59±106.46	0.02
2-n-butylacrylaldehyde	1070-66-2	79224.23±76975.77	91421.05±40717.15	63192.1±54725.76	3179.44±847.88	0.24
(Z)-6-nonenal	2277-19-2	99898.19±21732.49	33381.57±3437.91	94935.53±13920.62	11649.48±3949.83	<0.01
Acetamidoacetaldehyde	64790-08-5	104237.84±40340.56	102653.17±82189.49	7634.25±10.98	2348.44±751.18	0.04
3-Furaldehyde	498-60-2	6016.75±4181.55	1149.56±452.54	2954.97±1153.04	895.30±236.93	0.07
2,4-Decadienal	2363-88-4	6624.15±3192.97	1683.79±697.83	3340.43±220.49	2842.96±145.12	0.03
3-Hexenal	4440-65-7	4377.80±1807.34	263.22±124.06	2228.10±2143.58	615.05±32.09	0.03
Methacrolein	78-85-3	300.16±175.23	275.29±128.26	630.32±341.42	121.90±17.18	0.08
(E,E)-2,4-Octadienal	30361-28-5	3667.06±1320.60	1627.19±279.04	2326.82±102.96	1632.47±242.40	0.02
Hexanal	66-25-1	32678.83±6000.91	24930.22±4813.57	17811.66±1578.45	10468.50±1565.39	0.00
3-methylbutyraldehyde	590-86-3	97.08±7.17	413.41±327.19	9836.25±9811.78	107.09±83.85	0.10
(E)-Cinnamaldehyde	14371-10-9	416.11±77.43	259.52±33.46	280.1±28.76	276.25±57.08	0.02
(Z)-3-Hexenal	6789-80-6	5577.23±2595.45	1416.33±266.88	3588.82±2641.52	68.09±21.13	0.03
(E)-2-heptenal	18829-55-5	39833.42±9197.25	27630.34±3176.2	46614.58±17663.29	18904.57±5606.85	0.05

Compound name	CAS	AP	APHP	HP	HPAP	*P*-value
(E)-3,7-dimethyl-2,6-octadienal	141-27-5	282.26±61.7	61.91±8.45	111.48±92.08	55.74±75.81	0.01
(E,E)-2,4-heptadienal	4313-03-5	5500.62±2352.54	1868.8±298.69	3164.69±1132.82	1760.9±62.02	0.03
(Z)-2-decenal	2497-25-8	724.78±265.18	147.81±6.44	1219.89±642.4	75.4±10.74	0.01
(Z)-4-heptenal	6728-31-0	2309.01±2257.72	51.05±15.34	5.75±3.27	7.11±2.17	0.09
		595449.73	492542.15	546259.67	282584.46	
Alcohols（72 species）						
1-Pentanol	71-41-0	21810.7±2988.58	38948.58±3998.50	38808.65±20903.46	26194.31±11416.5	0.27
2-Propyl-1-heptanol	10042-59-8	88350.44±15772.73	69178.02±62136.07	86791.93±35450.08	18682.38±2061.85	0.15
β-Ethylphenethyl alcohol	2035-94-1	17.77±4.09	1625.75±733.68	1935.7±810.59	2186.68±256.22	0.01
4-hepten-1-ol	20851-55-2	4855.24±1926.39	1608.27±138.43	1858.65±470.26	2996.75±352.96	0.01
(E)-5-decen-1-ol	56578-18-8	101.61±44.73	449.94±52.55	329.54±24.64	711.98±147.25	<0.01
cis-1-methyl-4-(1-methylethyl)-2-cyclohexen-1-ol	29803-82-5	28.59±21.09	3145.32±1824.00	1930.71±866.32	2340.27±978.98	0.05
trans-2-Carene-4-ol	4017-82-7	89.81±69.09	5.13±3.04	37.81±31.32	1.32±1.24	0.07
2-Ren-1-enol	22104-79-6	3152.52±645.11	240.54±199.27	1046.59±140.91	300.92±83.34	<0.01
(E)-2-penten-1-ol	1576-96-1	973.81±271.78	391.12±108.52	574.45±46.55	628.98±74.06	0.01
2-methyl-1-butanol	137-32-6	24.99±13.43	27058.77±12182.24	160391±26621.07	33532.74±3544.51	<0.01
[1R-(1a,2a,3b6a)]-3-vinyl-3-methyl-2-(1-methylethenyl)-6-(1-methylethyl)cyclohexanol	35727-45-8	1613.96±465.40	133.98±41.74	255.10±124.21	149.57±125.20	<0.01
2-pentanol	6032-29-7	38253.81±6210.13	55980.87±12926.02	57298.59±12214.64	10124.21±5015.55	0.00
4-methyl-2-heptanol	56298-90-9	1039.03±175.98	2177.65±397.87	3443.89±197.10	1310.35±91.01	<0.01
2-phenoxyethanol	122-99-6	7564.09±2913.34	1419.12±627.92	3351.41±2130.83	2102.38±580.98	0.02
2-propen-1-ol	107-18-6	78.94±48.11	10546.84±9206.31	2082.14±1799.25	24085.49±8910.43	0.01

Compound name	CAS	AP	APHP	HP	HPAP	*P*-vau le
2-(2-butoxyethoxy)ethanol	112-34-5	11874.99±618.68	12204.24±1706.27	11907.46±866.92	11780.2±643.36	0.97
5-Hexen-1-ol	821-41-0	10370.43±1918.31	5292.12±4148.78	4681.91±3081.97	961.22±324.87	0.02
2-Hexanol	626-93-7	6.26±0.49	4.16±3	1765.44±2107.72	4.26±3.71	0.18
3-heptanol	589-82-2	791.31±125.19	756.43±247.05	1526.43±400.20	1208.61±207.00	0.02
6-methyl-5-hepten-2-ol	1569-60-4	1997.41±646.96	3860.05±356.79	3697.31±572.6	2498.68±531.18	0.01
2-methyl-1-pentanol	105-30-6	79160.08±15469.01	57301.61±9652.05	58999±5185.77	26670.41±6634.80	0.00
1,2-Propanediol	4254-15-3	7135.95±2899.53	4015.33±1764.42	12009.36±2955.99	2316.22±440.53	0.05
1-Tetradecanol	112-72-1	694.05±99.48	332.68±118.46	543.46±170.74	264.09±27.81	0.01
1-Hexanol	111-27-3	193367.33±42873.05	140458.77±18747.44	94452.35±4774.6	189933.57±23083.30	0.01
trans-3-Carene-2-ol		23.26±6.96	59.55±39.84	53.13±29.07	94.94±12.23	0.05
3-Methyl-3-buten-1-ol	763-32-6	19879.4±17168.89	584.6±578.88	11852.28±10252.92	35±33.84	0.11
2-Butanol	78-92-2	559.6±152.43	6.3±5.24	46.03±45.23	13.32±10.83	<0.01
2-Methyl-3-buten-2-ol	115-18-4	93.72±12.00	66.05±18.07	368.38±11.63	103.1±54.97	<0.01
benzyl alcohol	100-51-6	6913.97±1852.06	865.85±35.36	1148.15±77.58	988.64±57.53	<0.01
(Z)-3-Hexen-1-ol	928-96-1	1296.01±398.13	2838.8±2033.64	1936.38±1918.47	915.37±610.48	0.43
α,α,4-Trimethylbenzyl alcohol	1197-01-9	2367.71±1915.15	76445.76±25795.25	29691.56±12730.83	52235.51±16564.77	0.00
α,α,4-Trimethylcyclohexanemethanol	498-81-7	189.26±163.02	109.81±15.32	140.94±44.57	109.52±98.82	0.73
2-Undecanol	1653-30-1	12.98±7.02	15.28±8.18	18.17±12.3	13.98±9.46	0.91
1-Octen-3-ol	3391-86-4	319718.37±65273.15	109488.00±6179.30	298325.77±41738.3 2	36844.95±11452.35	<0.01
2,4-undecadien-1-ol	77657-78-4	141.45±104.28	129.58±7.39	33.01±15.92	104.02±28.59	0.15
1-Octanol	111-87-5	18889.19±7584.06	8475.32±2274.31	10672.00±7697.55	6584.87±2474.84	0.11
(R)-4-methyl-1-(1-methylethyl)-3-cyclohexe	20126-76-5	0.43±0.01	1.03±0.61	1.42±0.82	2.73±2.52	0.28

Compound name	CAS	AP	APHP	HP	HPAP	*P*-vaule
n-1-ol						
L-alpha-pinitol	10482-56-1	2323.68±711.82	662.12±277.87	1537.55±276.56	706.41±247.81	0.00
1-penten-3-ol	616-25-1	44.79±29.18	14743.85±4000.11	9129.04±7930.28	6221.54±4262.47	0.04
(1S-endo)-1,7,7-trimethylbicyclo[2.2.1]heptan-2-ol	464-45-9	46.77±20.42	21.03±5.92	54.9±42.24	29.05±8.64	0.35
6-undecanol	23708-56-7	4330.30±2235.59	2974.32±1867.58	2212.49±622.64	2437.83±830.61	0.39
(S)-3-ethyl-4-methylpentanol		5589.02±4453.28	2240.45±757.8	2389.72±557.01	3266.17±589.25	0.32
3-Hexen-1-ol	544-12-7	1469.78±224.98	4549.3±2298.32	2702.19±1088	1120.68±287.47	0.04
11-dodecenol	35289-31-7	11.30±7.60	3.55±2.62	18.57±16.77	7.31±8.58	0.38
2-octanol	123-96-6	5785.16±1250.91	8848.76±1560.71	14755.79±1916.34	7703.51±459.43	<0.01
(E)-2-nonen-1-ol	31502-14-4	4100±1259.63	2537.97±683.96	5003.12±1476.13	4210.3±441.54	0.10
(E,E)-2,4-heptadien-1-ol	33467-79-7	263.01±84.23	66.49±31.22	332.10±129.36	196.86±56.79	0.02
1-methoxy-2-propanol	107-98-2	22.41±17.08	825.05±534.17	11.38±4.41	7.02±1.66	0.01
Cyclohexanol	108-93-0	20.31±16.66	3020.7±157.16	4679.82±3166.26	3010.26±1791.11	0.07
1-heptanol	111-70-6	104291.06±36036.72	50511.2±15113.68	48823.45±9066.18	74245.31±11177.73	0.04
6,10,14-Trimethyl-2-pentadecanol	69729-17-5	7777.2±3205.38	4118.95±1789.77	12519.15±3182.07	2372.62±359.7	0.28
3-(methylthio)-1-propanol	505-10-2	14971.41±5246.07	3065.72±1091.29	1161.58±135.25	2087.11±358.61	0.01
3-methyl-1-butanol	123-51-3	709832.63±25183.31	375647.27±12127.60	105855.32±89314.28	329708.3±16604.60	<0.01
Ethanol	64-17-5	227892.43±61739.21	404497.00±18776.70	194834.65±157013.14	350335.60±59267.63	0.06
Juluanol	472-07-1	2.62±1.44	2.15±0.58	2.95±0.52	2.27±1.00	0.74
Isohumol	89-79-2	15.78±8.58	32.12±1.17	23.08±13.71	41.27±18.63	0.14

Compound name	CAS	AP	APHP	HP	HPAP	*P*-vaule
Tricyclo[5.2.1.0(1,5)]dec-5-en-8-ol		2504.57±118.42	1540.35±321.27	2743.84±745.58	964.97±528.39	0.01
(E)-2-tetradecen-1-ol		9.35±4.44	2485.66±959.08	4716.39±1701.88	1080.50±564.50	0.00
2-methyl-1-propanol	78-83-1	78.58±12.88	70.66±41.66	82.38±38.89	78.23±18.03	0.97
trans,cis-2,6-Ren-diene-1-ol	28069-72-9	5.94±2.37	19.32±9.14	10.41±9.87	8.82±8.01	0.27
1-butanol	71-36-3	243.09±245.67	6206.22±2527.14	8.87±7.41	1555.54±1536.11	0.00
2-methyl-6-hepten-1-ol		2014.21±664.66	3885.1±371.29	3745.04±568.29	2508.49±538.72	0.01
2-Ethyl-1-butanol	97-95-0	33.67±25.24	486.08±332.15	238.62±50.11	1228.32±370.08	0.00
isopropyl alcohol	67-63-0	226500.43±57321.34	12838.68±4079.21	134150.52±110912.08	418492.3±58285.90	0.00
(E)-2-hepten-1-ol	33467-76-4	4338.15±3814.67	2648.33±754.26	4432.65±152.94	3749.07±455.48	0.68
2-ethyl-1-hexanol	104-76-7	33897.9±16643.54	7399.64±1639.38	26373.71±14602.69	4520.89±1961.03	0.03
(-)-cis-isopiperonyl menthol	96555-02-1	2.55±0.54	5.71±3.55	7.75±3.83	8.01±2.96	0.18
cis-Verbenzyl alcohol	1845-30-3	8.46±6.67	1.5±1.26	7.07±11.46	1.65±0.83	0.49
3-octanol	589-98-0	13562.46±5238.27	92612.89±37726.41	180368.1±72052.34	139154.97±33521.51	0.00
1-methyl-4-(1-methylethyl)cyclohexanol	21129-27-1	2189.54±613.18	930.94±267.11	1113.38±904.01	569.02±180.11	0.04
5-methyl-2-(1-methylethyl)cyclohexanol	1490-04-6	119.63±73.7	74.08±63.92	80.83±67.19	19.61±18.08	0.31
cis-Linalool oxide		728.16±123.96	505.38±152.54	613.36±140.22	402.59±93.31	0.07
		2218464.82	1646299.76	1668745.87	1821083.94	
Ester (52 species)						
4-Nitrophenyl 3-methylbut-2-enoate		2183.59±1385.66	1628.48±346.34	2022.01±342.57	1631.15±134.92	0.73
Bis(2-methylpropyl) phthalate	84-69-5	169.06±75.57	12.25±8.93	95.08±52.16	90.69±64.41	0.06
Allyl nonanoate	7493-72-3	498.95±189.78	2324.23±2001.38	150.12±45.59	353.89±144.6	0.10
Methyl Butyrate	623-42-7	2156.06±1076.87	17.12±3.55	28.57±16.43	15.19±5.21	0.00

Compound name	CAS	AP	APHP	HP	HPAP	*P*-vau le
Ethyl linoleate	544-35-4	422.27±79.69	1569.42±658.42	679.77±337.5	1331.08±424.08	0.04
Ethyl 9-Octadecenoate	6512-99-8	236.59±31.98	15.42±13.28	214.23±146.8	182.62±104.95	0.07
Ethyl Decanoate	110-38-3	120.2±79.06	4406.43±2010.48	434.17±138.25	2614.26±1079.70	0.00
2-Tetradecyl Methoxyacetate		43.11±25.99	1157.85±955.05	689.35±658.63	421.12±285.60	0.22
Thio S-Methyl Octanoate	2432-83-9	39.26±25.05	5162.28±232.09	94317.25±41403.35	69731.42±58214.60	0.54
Vinyl propionate	105-38-4	258.9±69.69	132.39±57.32	9821.86±1281.02	361.97±295.44	0.01
S-Methyl Propyl Thioester	5925-75-7	20808.88±11173.03	43809.83±14507.99	11624.25±10414.69	18273.43±6184.64	0.03
Ethyl 2-hydroxybenzoate	118-61-6	98.14±69.89	96.42±38.42	1052.58±129.27	293.73±108.96	<0.01
Ethyl pentyl phthalate		3440.58±370.09	5974.19±3727.01	4710.68±1461.74	5938.33±2360.27	0.53
Propyl propionate	106-36-5	26.88±24.43	11727.17±4243.43	675.34±285.32	3224.3±1815.77	0.00
2-Hydroxy-Y-butyrolactone	19444-84-9	457.49±447.61	18.09±10.29	71.73±69.49	12.62±1.78	0.12
Isopropyl myristate	110-27-0	49.41±27.04	7.74±3.60	5.55±3.95	5.61±0.30	0.01
2-Phenylethyl Propionate	122-70-3	7211.78±1290.04	10717.05±3113.38	7885.14±1757.4	8537.55±992.12	0.23
Ethyl acetate	141-78-6	3421.18±1504.50	8591.44±7730.20	1700.05±565.13	7429.5±3297.14	0.24
Ethyl 3-methylbutyrate	108-64-5	47.4±0.19	65703.33±21328.26	2327.24±1017.01	167666.17±39067.20	<0.01
Methyl 9-octadecenoate	2462-84-2	13.86±10.46	222.51±186.07	843.77±100.14	4.83±4.37	<0.01
Methyl diethylcarbamate	686-07-7	3005.60±328.94	2236.20±444.35	2206.71±179.18	2559.87±440.67	0.09
Hexyl acetate	142-92-7	2988.56±2663.56	150.85±141.09	56.65±0.84	20.17±16.88	0.07
Ethyl 2-hydroxypropionate	97-64-3	9591.29±2125.19	2713.38±1038.21	1968.51±361.9	3416.1±213.17	<0.01
Ethyl alpha-methylphenylpropionate	34666-01-8	258.26±64.54	2.19±1.71	64.78±25.29	1.26±0.91	<0.01
Allyl Acetate	591-87-7	112491.99±37104.88	62836.56±6669.45	53.85±48.46	10.80±14.54	<0.01
Methyl methacrylate	80-62-6	85.79±74.20	12.49±9.73	24.15±14.94	21.11±16.08	0.16
Bornyl acetate	76-49-3	3713.90±1457.41	1836.97±210.43	2468.26±810.49	1709.95±227.92	0.07

Compound name	CAS	AP	APHP	HP	HPAP	*P*-vaule
exo-1,7,7-trimethylbicyclo[2.2.1]hept-2-yl 2-propenoate	5888-33-5	380.25±155.20	27.39±14.39	290.66±321.22	39.59±19.38	0.10
Thio S-methylbutyrate	2432-51-1	295.40±177.62	4711.21±2231.14	1851.87±1612.7	1550.35±1176.22	0.04
Vinyl acetate	108-05-4	5191.87±470.55	21145.94±30700.86	14783.73±12859.38	474.53±98.69	0.47
Linalyl acetate	115-95-7	5.67±6.68	5.49±3.61	11.53±2.37	8.13±6.3	0.47
Methyl salicylate	119-36-8	1405.39±522.57	386.57±361.41	923.93±656.7	204.02±126.09	0.05
Methyl 3-furancarboxylate	13129-23-2	51153.04±11378.01	47639.23±29655.64	45752.13±18711.06	10587.62±8673.99	0.10
Methyl 2-methyl-2-butenoate	41725-90-0	31099.28±18314.25	353.80±199.07	2.06±1.06	24602.56±1235.82	0.01
Dibutyl Cold Benzene Dicarboxylate	84-74-2	4317.77±682.68	4020.22±746.85	4619.87±995.33	4999.46±1683.32	0.73
Ethyl formate	109-94-4	12925.72±10068.96	19.11±0.64	14.66±1.12	10.04±8.23	0.03
Isopropyl acetate	108-21-4	3421.18±1504.50	11549.4±4046.12	1791.16±411.16	8374.41±3471.12	0.01
1-Octen-3-yl acetate	2442-1-6	671.09±594.47	189.51±146.35	509.29±441.39	12.44±2.77	0.21
Octanoic acid formate	112-32-3	45428.12±4250.03	26135.71±14252.27	20160.62±15119.72	25165.38±5317.46	0.09
Ethyl (E)-2-butyrate	623-70-1	1.33±0.99	12.89±1.7	207.28±242.05	3.49±0.09	0.18
Ethyl 2-methylbutyrate	7452-79-1	1074.74±362.64	1614.76±706.48	11869.22±3001.71	3244.85±1329.9	<0.01
2,2,4-Trimethyl-1,3-pentanediol diisobutyrate	6846-50-0	649.96±368.83	174.26±42.28	328.16±269.44	131.49±56.43	0.09
3-Methyl-1-Butyl Acetate	123-92-2	13352.23±2826.81	1503.08±874.39	1774.62±864.97	1623.29±317.52	<0.01
Hexyl butyrate	2639-63-6	9959.35±1017.55	3629.07±323.78	2962.49±661.66	1922.05±139.69	<0.01
1-Methyl-4-(1-methylethenyl)cyclohexanol acetate	10198-23-9	149580.9±36067.3	99865.94±94424.36	69941.31±18072.51	1031.15±485.91	0.04
n-propyl acetate	109-60-4	95825.84±3707.85	58167.33±9411.74	28423.77±7205.98	549.49±77.89	<0.01
3-Methylbutanoic acid	1118-27-0	175.59±35.29	48.76±28.35	148.14±59.22	19.78±14.94	0.00

Compound name	CAS	AP	APHP	HP	HPAP	*P*-vaule
1-vinyl-1,5-dimethyl-4-hexenyl ester						
Methyl phenylacetate	101-41-7	818.68±685.49	2096±507.49	2729.32±2345.42	87.29±17.80	0.11
11-Dodecen-1-yl acetate	35153-10-7	416.47±375.32	102.85±77.20	208.94±60.41	49.34±34.40	0.18
3-Methylbutyl 2-methylpropanoate	2050-01-3	23.63±22.80	14462.29±5830.91	8439.19±6341.49	9213.36±2320.04	0.03
		602012.48	530943.09	363935.6	389762.83	
Ketones（47 species）						
2-Methyldihydro-3(2H)-thiophenone	13679-85-1	2564.73±598.71	21436.12±4304.17	18758.43±1251.13	4384.88±995.56	<0.01
2-Heptanone	110-43-0	47653.26±14269.56	334.89±75.67	619.07±288.21	82.36±18.41	<0.01
Acetone	67-64-1	64421.91±2167.04	48516.14±30791.67	107684.51±42597.78	126184.27±14308.04	0.03
4-Octanone	589-63-9	1161.04±862.93	1.46±0.68	2.24±0.67	295.82±363.73	0.05
3,3-Dimethyl-2-butanone	75-97-8	108761.38±46991.67	59729.57±19848.84	60502.43±17923.00	39049.35±8078.50	0.07
Cyclohexanone	108-94-1	5062.46±4253.98	17.61±3.75	377.82±359.2	1136.25±510.32	0.07
2,3-dimethyl-2-cyclopenten-1-one	1121-05-7	8.78±1.43	13.47±3.68	4.75±4.26	5.39±1.99	0.03
(E)-6,10-dimethyl-5,9-undecadien-2-one	3796-70-1	3746.77±1403.79	2074.98±1681.35	2279.85±1114.76	1201.38±485.96	0.17
5-Hexanedihydro-2(3H)-furanone	706-14-9	922.44±56.31	1063.21±782.48	1011.71±812.05	2278.31±771.41	0.12
2-Pentadecanone	2345-28-0	7.39±1.04	0.89±0.79	0±0.01	12.95±10.26	0.05
1-cyclododecylethanone	28925-00-0	567.38±423.41	407.33±128.88	465.37±53.89	437.42±78.21	0.84
2-Decanone	693-54-9	430.61±118.83	1674.44±1360.81	2776.96±2295.92	1570.67±97.66	0.28
6-pentyltetrahydro-2H-pyran-2-one	705-86-2	724.27±34.6	176.9±159.75	160.16±139.44	138.45±6.6	<0.01
5-methyl-4-hexen-3-one	13905-10-7	2406.84±2132.41	24.9±23.17	3478.87±1294.17	217±205.92	0.02
2,6-di-tert-butyl-4-hydroxy-4-methylcyclohexa-2,5-dien-1-one	10396-80-2	19.73±10.12	72.73±34.51	76.88±24.12	20.70±9.52	0.02

Compound name	CAS	AP	APHP	HP	HPAP	*P*-vaule
3-Octanone	106-68-3	131102.14±96714.86	168745.90±8290.79	274564.50±23213.00	75262.30±38866.45	0.01
1-hydroxy-2-propanone	116-09-6	3.04±0.6	221.18±67.73	325.07±330.37	752.35±765.07	0.24
4,6,6-trimethylbicyclo[3.1.1]hept-3-en-2-one	80-57-9	36.74±13.38	48.65±25.6	74.13±41.14	99.98±39.85	0.15
4-hydroxy-4-methyl-2-pentanone	123-42-2	343.92±144.49	8094.6±763.19	1379.87±1315.54	10440.03±5096.64	0.00
2-Renone	821-55-6	109834.58±28685.51	73189.83±29311.78	35971.00±10334.05	27300.15±8910.79	0.01
3-methyl-2,5-furandione	616-02-4	10.24±2.75	1.47±1.17	72.18±15.12	66.07±53.4	0.03
6,10-dimethyl-5,9-undecadien-2-one	689-67-8	13910.12±3328.12	9959.73±2578.92	11094.62±618.01	5939.64±535.03	0.01
Isophorone	78-59-1	4231.96±2108.69	1996.68±296.32	4735.23±3474.04	713.88±519.37	0.13
1-(4-methylphenyl)-1-pentanone	1671-77-8	126.28±104.19	80.31±29.79	162.46±60.26	23.62±2.72	0.11
1-Octen-3-one	4312-99-6	2939.00±761.89	284.60±199.61	1915.91±387.82	662.95±221.00	<0.01
2(5H)-furanone	497-23-4	4119.73±2408.1	598.34±492.48	2.88±1.40	20.7±3.63	0.01
acetophenone	98-86-2	15973.23±1709.17	12393.31±1145.53	13552.98±1478.06	11637.55±744.53	0.02
6-methyl-5-hepten-2-one	110-93-0	3600.44±948.77	24.71±21.61	1412.53±1037.69	21.07±16.35	0.00
3-methyl-2-hexanone	2550-21-2	13470.3±2890.48	315.32±126.22	1228.86±1281.31	1224.1±1008.85	<0.01
5-pentyldihydro-2(3H)-furanone	104-61-0	3314.95±494.39	2884.76±371.61	5050.78±936.74	3758.32±664.52	0.02
5-Ethyldihydro-2(3H)-furanone	695-06-7	2746.33±368.57	5210.39±477.62	4721.27±463.19	1780.15±953.07	<0.01
6-propyltetrahydro-2H-pyran-2-one	698-76-0	433.48±51.62	110.15±17.82	146.93±27.58	49.36±20.93	<0.01
3-Methyldihydro-2(3H)-furanone	1679-47-6	2330.2±313.53	0.66±0.22	0.93±0.07	11.23±15.81	0.10
2-Ren-4-one	32064-72-5	10385.45±8708.16	6042.55±558.63	13262.05±803.46	3578.22±924.5	0.10
5-butyldihydro-2(3H)-furanone	104-50-7	1986.20±344.30	1947.17±271.90	2001.39±267.18	1424.95±306.62	0.13
3,5,-Octadien-2-one	38284-27-4	2430.31±655.48	1242.41±118.25	1485.6±214.66	1154.62±61.06	0.01
(-)-Carvone	6485-40-1	156.56±7.84	699.96±419.42	960.77±922.49	1778.01±468.71	0.04

Compound name	CAS	AP	APHP	HP	HPAP	*P*-vaule
1-(1H-pyrrol-2-yl)-ethanone	1072-83-9	11072.69±5132.94	203862.4±21077.4	1183251.67±97282.39	645692.57±237120.26	<0.01
3-Hexanone	589-38-8	355.12±200.49	380.40±380.35	213.62±7.51	6.17±0.33	0.21
(E,E)-3,5-octadien-2-one	30086-02-3	10389.88±3267.77	4771.99±702.47	7452.29±369.72	4572.86±163.95	0.01
2-Octanone	111-13-7	28475.55±17104.69	1508.57±421.86	2649.32±2022.27	6364.8±891.17	0.02
5-Methyl-2-hexanone	110-12-3	8.36±3.78	10961.13±2353.04	121.08±24.01	3584.33±3051.16	<0.01
2-Undecanone	112-12-9	28038.47±4445.12	86054.11±41156.01	120776.53±77832.38	107382.3±36854.79	0.17
2,3-Hexanedione	3848-24-6	265.71±221.66	14930.35±7089.99	8537.24±7462.61	9496.06±3249.95	0.06
3-Hexen-2-one	763-93-9	106.95±106.71	8665.02±7071.68	20523.17±18373.08	20201.5±1750.48	0.10
2,3-Octanedione	585-25-1	513.85±244.37	1270.41±228.06	39505.5±34175.09	6045.76±8376.26	0.08
3-Pentanone	96-22-0	86286.92±25650.95	15205.45±4814.99	22424.8±1180.6	6690.85±5441.13	<0.01
		727457.69	777247.15	1977776.21	1134751.65	
Olefins（43 species）						
α-Dehydro-cedrene	78204-62-3	3.47±0.43	3.11±2.33	3.14±1.86	5.12±2.25	0.54
α-Guaiacene	654486	1.36±0.50	1.84±0.74	1.95±1.07	0.72±0.58	0.15
Isochlorophyllene	95910-36-4	5.69±1.77	48.97±30.21	88.87±75.83	3.81±1.64	0.10
Cubanene	29837-12-5	6±3.54	4.2±3.58	2.49±0.36	2.33±1.50	0.35
(E)-1,3-rutadiene	56700-77-7	1690.39±770.22	1711.3±819.96	3635.58±424.4	2310.45±67.47	0.01
4-Methyl-1-decene	13151-29-6	191.62±297.26	7927.66±5943.78	5998.4±3587.74	24139.02±3985	<0.01
1-Pentadecene	13360-61-7	181.3±129.46	102.16±28.98	426.5±284.67	109.55±101.81	0.13
Humulene	6753-98-6	1568.89±179.02	809.03±58.82	446.78±52.47	98.5±42.52	<0.01
A-pinene	80-56-8	1747.31±35.52	378.68±334.29	1343.46±1085.49	595.38±481.91	0.08

Compound name	CAS	AP	APHP	HP	HPAP	*P*-vaule
Camphene	79-92-5	1.60±1.54	127.67±20.55	2731.05±2591.16	1574.06±1257.79	0.14
(E)-β-farnesene	18794-84-8	141.23±32.98	18.29±4.44	15.72±14.53	11.83±4.66	<0.01
(-)-3,7,7-Trimethyl-11-methylene-spiro[5.5]undec-2-ene	18431-82-8	28.49±24.70	2291.36±1394.52	324.87±93.29	358.61±247.25	0.02
(Z)-4-tridecatriene	41446-54-2	2350.83±1883.06	13.42±3.86	2.86±1.56	2.53±0.38	0.04
2,4,6,8-tetramethyl-1-1-undecatriene	59920-26-2	7577.46±6559.8	1157.18±416.57	1928.88±1575.97	188.29±168.33	0.10
hexadecene	629-73-2	39487.24±6039.08	15584.46±6563.47	18290.05±3801.01	13577.39±1852.25	0.00
5-ethyl-1,3-cyclohexadiene	40085-08-3	754.98±167.28	89.31±59.78	229.29±8.6	82.15±23.61	<0.01
bicyclo[4.1.0]hept-2-ene	2566-57-6	0.34±0.24	3.74±2.46	4.56±3.44	3.94±3.59	0.31
cis-alpha-limonene	18252-46-5	5.84±3.85	6.68±3.19	7.91±3.66	8.47±2.74	0.78
alpha-Hydrocotylene	99-83-2	784.22±214.51	20988.3±3892.16	11879.76±10045.42	17002.26±6436.86	0.02
R(-)3,7-dimethyl-1,6-octadiene	10281-56-8	308.00±248.08	12.52±4.97	1365.09±710.16	273.47±95.98	0.01
(Z)-3-octene	14850-22-7	34744.67±11740.19	20115.65±714.09	21808.45±18056.57	19531.42±7119.03	0.37
10,11-epoxycarbene	143785-42-6	4.21±0.03	2.46±0.73	4.14±2.1	3.12±1.77	0.42
1-pentene	109-67-1	4328.7±3138.33	117.43±195.96	145.12±74.92	7777.8±4271.54	0.02
(1R)-2,6,6-trimethylbicyclo[3.1.1]hept-2-ene	7785-70-8	1466.59±529.63	945.36±329.18	1649.92±374.62	482.52±408.89	0.03
(S)-1-methyl-4-(1-methylethenyl)cyclohexene	5989-54-8	4.17±0.40	691.99±598.69	425.15±378.18	10522.4±7709.7	0.03
Y-pinene	99-85-4	496.64±487.45	175.2±119.89	297.24±28.6	2.93±2.18	0.19
(Z)-3,7-dimethyl-1,3,6-octatriene	3338-55-4	128.32±104.41	2246.56±788.73	3005.77±1017.57	817.42±722.84	0.01
1-methyl-4-(1-methylethylidene)cyclohexene	586-62-9	666.68±356.24	20.66±0.35	804.42±467.64	182.02±266.23	0.05
alpha-Caracole	21391-99-1	13.19±7.58	9.20±2.85	10.61±2.81	11.85±1.71	0.72

Compound name	CAS	AP	APHP	HP	HPAP	*P*-vaule
3-Carene	13466-78-9	2792.97±2540.02	170.42±102.59	976.66±382.95	196.84±83.67	0.11
bicyclo[4.2.0]octa-1,3,5-triene	694-87-1	801.36±800.94	102699.39±15138	174212.37±51042.73	70015.51±26609.7	0.00
trans-3-methyl-6-(1-methylethyl)cyclohexene	1124-26-1	2.32±2.15	12.13±4.52	13.72±6.37	10.13±10.84	0.25
(E,Z)-2,6-dimethyl-2,4,6-octatriene	7216-56-0	20.27±16.46	13.42±10.34	105.03±72.81	48.82±14.8	0.07
Dimethyldiazene	503-28-6	149448.4±118563.03	8631.83±7780.05	187326.03±22739.76	126898.07±16487.7	0.03
(E)-5-octadecene	7206-21-5	3744.59±899.55	67495.09±23020.78	28680.7±12060.37	45120.78±14466.73	0.01
2,3,6-Trimethyl-1,5-heptadiene	33501-88-1	31.80±7.47	74.71±53.31	21.05±14.27	43.82±29.16	0.26
β-Terpinene	99-85-4	113651.59±37011.52	14414.6±11983.76	154459.57±6390.44	20614.47±28373.47	<0.01
trans-β-Terpinene	3779-61-1	5.45±2.38	19.1±13.82	91.6±87.79	11.06±9.45	0.14
Laurylene	123-35-3	31.24±13.15	19.03±13.23	303.34±279.4	242.2±204.81	0.18
Limonene	138-86-3	330037.35±81603.55	64.99±24.59	3482.98±3290.36	104.69±46.15	<0.01
4-Acetoxy-3-methoxystyrene	46316-15-8	242.49±27.64	168.83±33.76	233.19±28.33	168.28±28.82	0.02
1-methyl-4-(1-methylethyl)-1,3-cyclohexadiene	99-86-5	652.89±475.57	188979.9±13969.3	121017.19±104761.68	101373.85±93410.52	0.06
2,6-dimethyl-2,4,6-octatriene	C10H16	3.84±3.58	0.81±0.70	1328.19±1096.52	2.13±0.65	0.04
		700155.99	458368.64	749129.65	464530.01	
Alkanes（19 species）						
2-Methylne	3221-61-2	64488.28±2156.68	54476.2±27403.16	105990.14±40905.23	125305.37±13301.62	0.03
Trichloromethane	67-66-3	125669.90±10862.51	89653.48±6193.53	83073.76±64555.07	46180.1±39170.52	0.17

Compound name	CAS	AP	APHP	HP	HPAP	*P*-vaule
Undecane	1120-21-4	1084.32±259.65	278.18±159.02	4604.55±3868.78	5325.47±4760.84	0.19
3,7-Dimethylrenane	17302-32-8	30354.97±26489.37	2001.2±95.14	73903.93±69863.47	48513.00±8381.18	0.20
2,9-Dimethyldecane	1002-17-1	499.52±432.44	48.81±5.05	138.23±134.24	13.75±10.60	0.10
4,8-dimethylundecane	17301-33-6	25.31±0.49	11.66±7.84	10575.29±9546.57	1739.63±795.88	0.08
7,7-dimethyl-2-methylenebicyclo[2.2.1]heptane	471-84-1	35199.47±29342.38	343726.43±38251.19	446588±46678.7	314887.8±70143.9	<0.01
Decane	124-18-5	89249.2±5857.13	44024.97±19527.87	63761.23±10249.66	50074.27±5633.41	0.01
Cyclotetradecane	295-17-0	15.71±8.86	1090.22±176.08	15.64±6.24	0.37±0.03	<0.01
1,2-Dichloroethane	107-06-2	882.11±91.96	820.62±117.15	1804.2±389.45	801.58±118.15	0.001
3-Ethyl-3-methylheptane	17302-01-1	0.57±0.01	0.55±0.05	2.46±1.33	27.87±13.61	0.003
1,1-Diethoxyethane	105-57-7	45.09±16.36	5567.1±5551.25	1068.14±607.56	518.89±504.95	0.136
Diethoxymethane	462-95-3	3889.93±2509.22	7863.11±5201.66	2672.55±2381.84	2023.67±871.9	0.183
1-methyl-4-(1-methylethyl)-7-oxabicyclo[2.2.1]heptane	470-67-7	4.45±0.09	15606.66±7620.64	11302.92±5792.7	7558.73±6517.15	0.054
5-methyldecane	13151-35-4	241.39±232.44	23505.31±18296.67	31267.52±13825.19	64667.42±54221.11	0.137
4-methylene-1-(1-methylethyl)bicyclo[3.1.0]hexane	3387-41-5	40.88±27.07	12.11±9.88	18.34±12.95	27.65±30.3	0.449
3-methyleneheptane	1632-16-2	5297.84±486.17	5073.96±814.79	4825.25±477.93	5706.24±999.17	0.533
1,7,7-trimethyltricyclo[2.2.1.0]heptane	508-32-7	42.08±24.92	46.96±39.18	51.41±20.42	27.16±20.39	0.727
Spiro[cyclopropane-1,6`-[3]oxatricyclo[3.2.1.0(2,4)]octane]	107079-37-8	1171.86±514.97	433.92±212.92	980.75±120.1	659.45±11.64	0.052
		358202.88	594241.45	842644.31	674058.42	
Aromatic compounds（31 species）						

Compound name	CAS	AP	APHP	HP	HPAP	*P*-vaule
1-(1,5-Dimethyl-4-hexenyl)-4-methylbenzene	644-30-4	2.49±1.72	0.9±0.18	6.02±2.38	1.87±2.01	0.036
1,4-Dichlorobenzene	106-46-7	1897.44±242.91	1000.82±205.22	1810.25±644.73	605.5±286.67	0.009
p-Xylene	106-42-3	28502.35±10147.14	10965.24±1791.11	21951.2±3029.07	709.47±99.15	0.001
Biphenyl	92-52-4	491.38±61.87	401.36±43.39	453.78±222.83	227.32±196.83	0.236
Pentylbenzene	538-68-1	3747.77±951.46	2704.92±1045.24	4434.67±1559.68	5595.47±4714.18	0.598
1-methoxy-2-methylbenzene	578-58-5	2166.41±2047.45	2745.43±676.76	4468.10±159.43	2898.1±630.52	0.155
Toluene	108-88-3	34696.13±1324.02	29018.45±4336.64	46343.10±6940.21	28405.98±4545.95	0.006
[3aS-(3aa,3bb,4b,7a,7aS*)]-7-methyl-3-methylene-4-(1-methylethyl)octahydro-1H-cyclopenta[1,3]cyclopropa[1,2]benzene	13744-15-5	41.9±18.17	22.97±0.98	30.69±15.1	18.67±13.18	0.244
(1,1-dimethylpropyl)benzene	2049-95-8	819.02±488.71	1782.02±585.13	1597.31±498.08	2976.24±349.29	0.004
homotrimethylbenzene	108-67-8	183.84±26.89	2436.2±514.77	1768.28±1542.27	748.05±633.11	0.051
Propylbenzene	103-65-1	1.53±0.80	867.64±747.89	17.08±15.24	292.92±292.66	0.092
ethylbenzene	100-41-4	28087.31±2080.05	19093.67±3340.39	32168.21±9472.59	13112.75±3762.15	0.01
n-Butylbenzene	104-51-8	2104.64±1898.68	2040.05±1004.67	1084.25±886.64	167.11±124.42	0.215
o-Xylene	95-47-6	18889.28±16389.41	14163.13±12424.03	20694.23±19783.49	199.41±55.89	0.349
1,2-Dichloro-3-methylbenzene	32768-54-0	1133.29±933.52	753.44±146.43	1433.86±625.75	131.15±101.75	0.1
1,3-Dimethylbenzene	108-38-3	47678.3±15736.92	25049.9±8933.86	35789.21±6533.63	14381.5±4166.6	0.017
1-Vinyl-3,5-dimethylbenzene	5379-20-4	4757.14±3901.2	4951.58±1037.67	4595.4±1097.86	1952.52±624.21	0.327
benzene	71-43-2	1474.27±20.69	861.31±769.73	1565.81±516.07	3366.25±2237.12	0.145
(R)-1-methyl-4-(1,2,2-trimethylcyclopentyl)benzene	16982-00-6	4.33±1.77	1.84±1.15	2.33±0.94	1.34±0.99	0.08

Compound name	CAS	AP	APHP	HP	HPAP	*P*-vaule
1,4-dichloro-2-methylbenzene	19398-61-9	9.93±4.05	739.72±192.75	1159.28±988.97	340.08±214.35	0.109
1-ethyl-3,5,-dimethylbenzene	934-74-7	231.06±54.06	86.26±77.85	73.32±39.61	36.35±32.54	0.01
2-methylnaphthalene	91-57-6	1974.71±211.7	1460.33±189.82	1977.49±661	1339.1±238.74	0.147
[1S-(1a,4ab,8aa)]-1-(1-methylethyl)-4,7-dimethyl- 1,2,4a,5,6,8a-hexahydronaphthalene	24406-05-1	1.52±0.72	1.55±1.17	2.10±0.49	1.45±0.38	0.709
(1a,4ab,8aa)-7-methyl-4-methylene-1-(1-methylethyl)1,2,3,4,4a,5,6,8a-octahydronaphthalene	39029-41-9	5.25±1.56	3.91±0.73	2.33±0.63	4.79±3.01	0.265
1,6-dimethyl-4-(1-methylethyl)naphthalene	483-78-3	119.64±101.4	4.84±1.30	89.1±84.32	109.91±41.48	0.241
1,2,3,4,4a,7-hexahydro-1,6-dimethyl-4-(1-methylethyl)naphthalene	16728-99-7	7.91±3.19	3.6±2.91	2.34±0.64	4.97±2.28	0.106
(1S-cis)-1,2,3,4-tetrahydro-1,6-dimethyl-4-(1-methylethyl)naphthalene	483-77-2	33.67±22.29	37.3±29.52	23.74±22.29	13.1±6.69	0.556
[1aR-(1aa,4a,4ab,7ba)]-1,1,4,7-tetramethyl-1a,2,3,4,4a,5,6,7b-octahydro-1H-cycloprop[e]azulene	489-40-7	164.81±59.67	114.57±68.48	190.03±20.14	144.83±28.15	0.342
azulene	275-51-4	15358.82±2065.01	10761.15±1884.53	15586.61±5472.14	9995.88±2668.73	0.15
P-cymene	99-87-6	411.87±269.37	3066.96±2356.21	11922.79±2051.08	636.61±150.99	<0.01
Indene	496-11-7	684.33±417.86	656.62±132.64	847.41±274.9	236.38±156.53	0.11
		195682.34	135797.68	212090.32	88655.07	
Acids（9 species）						
3-Methylbutyric acid	503-74-2	108073.49±26019.92	108990.81±114040.82	110744.96±66740.53	8908.47±2210.98	0.25

Compound name	CAS	AP	APHP	HP	HPAP	*P*-vaule
2-Methylbutyric acid	116-53-0	26195.46±11481.44	8098.39±5706.02	22924.92±14639.18	5729.67±193.58	0.088
E-11-tetradecenoic acid		25.88±5.79	35.56±4.07	38.03±15.14	28.76±6.66	0.37
Formic acid	64-18-6	11178.65±3766.55	51272.21±19345.13	98660.88±40149.62	57725.32±6820.88	0.01
Butyric acid	107-92-6	8490.41±1494.47	8090.97±3771.8	6580.25±2208.27	6034.58±1582.8	0.579
Oxalic acid	144-62-7	20.52±16.33	14.18±12.83	632.37±211.43	28.94±50.13	<0.01
(R)-(-)-4-methylhexanoic acid	52745-93-4	457.99±373.46	288.7±17.06	167.57±71.51	296.13±80.44	0.395
Heptanoic acid	111-14-8	3686.24±361.43	3412.41±1153.28	3287.07±875.60	3202.92±436.08	0.879
Acetic acid	64-19-7	42549.95±36435.69	17155.42±25891.77	3669.98±3201.64	43005.82±37818.37	0.332
heterocyclic（19 species）						
2-Hexylfuran	3777-70-6	2744.42±555.41	2467.04±667.67	2187.8±791.63	1613.95±741.88	0.299
2-Ethylpyridine	100-71-0	4879.92±688.95	2373.59±552.13	4977.74±213.2	2116.53±374.74	<0.01
2-Butylfuran	4466-24-4	4840.52±1366.40	2823.68±854.82	4151.26±543.43	1845.75±1580.70	0.152
Trimethylpyrazine	14667-55-1	5962.18±779.15	8139.75±3528.17	12018.97±922.33	7318.41±1242.8	0.027
2,3-Dihydrofuran	1191-99-7	68.58±54.55	1768.50±1637.78	468.88±530.66	11.77±10.96	0.115
Methyl pyrazine	109-08-0	909.40±90.82	420.19±20.18	1809.73±55.82	978.29±50.20	<0.01
2,3-Dimethylpyrazine	5910-89-4	134.53±116.22	46.44±1.87	764.91±27.79	91.62±45.75	<0.01
3-Ethylpyridine	536-78-7	1495.28±111.26	1359.77±171.44	4504.87±337.25	2095.89±278.97	<0.01
2-ethylfuran	3208-16-0	7220.75±2061.01	6089.95±1946.16	9105.5±609.86	5835.39±797.98	0.099
2-(methoxymethyl)furan	13679-46-4	18.20±9.46	5486.82±3678.29	7243.15±6573.85	80.46±69.71	0.1
trans-2-(2-pentenyl)furan	70424-14-5	684.43±294.72	140.46±39.30	823.41±469.00	237.27±88.46	0.046
2,4-dimethylfuran	3710-43-8	2319.74±1489.55	2001.69±1082.38	2092.67±1249.69	1120.35±146.98	0.595
2,5,-dimethylpyrazine	123-32-0	69204.11±23504.08	34180.18±1848.35	73364.84±43543.86	29450.14±12856.57	0.142
2-Pentylfuran	3777-69-3	45278.82±20551.54	1817.81±2806.19	2174.45±1650.98	313.84±171.96	0.002

Compound name	CAS	AP	APHP	HP	HPAP	*P*-vaule
2-methylfuran	534-22-5	3.95±2.65	45.89±38.43	5.57±5.95	397.91±324.05	0.049
pyrrole	109-97-7	694.81±421.72	94.35±86.5	53.26±35.03	113.06±113.64	<0.01
3-Methyl-2,3-dihydrobenzofuran	13524-73-7	101.49±100.97	2.03±0.57	219.85±212.60	12.47±11.74	0.165
trans-Linalool Oxide (furan type)	34995-77-2	609.49±113.00	323.69±181.06	506.98±96.38	351.95±72.41	0.065
Indole	120-72-9	17156.53±13214.43	24417.96±680.43	349518.67±128830.06	303492.23±86653.80	0.001
Phenolic compounds（10 species）						
2,6-Dimethylphenol	576-26-1	5503.52±1036.84	1694.51±996.02	1088.8±180.28	2133.03±257.31	<0.01
3-Methylphenol	108-39-4	3470.65±424.58	3382.35±574.13	4172.71±198.55	4697.57±493.02	0.02
8,9-Dehydrothymol	18612-99-2	17.34±1.83	10.33±6.85	47.21±31.79	17±13.16	0.121
Methyl eugenol	93-15-2	2.2±0.14	1.83±0.22	1.14±0.26	1.04±0.19	<0.01
Eugenol	97-53-0	2.92±1.86	0.53±0.29	3.74±1.69	0.56±0.62	0.034
3-Ethylphenol	620-17-7	728.85±128.05	350.97±86.24	322.35±139.12	266.12±29.66	0.002
Thymol	89-83-8	199.93±50.34	146.76±20.64	151.83±18.06	127.71±15.98	0.084
2-Methylphenol	95-48-7	146.82±9.22	172.91±30.54	163.01±26.92	193.4±10.79	0.138
4-Ethyl-2-methylphenol	2219-73-0	237.30±232.43	53415.10±9994.9	37194.16±32187.15	30061.37±27152.32	0.085
3,5-Bis(1,1-dimethylethyl)phenol	1138-52-9	1444.83±142.10	1092.07±61.84	1478.12±392.72	1129.07±206.09	0.159
Other compounds（17 species）						
1-Tetradecynyl	765-10-6	10434.46±2502.51	59.81±16.78	2547.53±942.47	30.05±8.43	<0.01
Dimethyl tetrasulphide	5756-24-1	46.84±29.00	2006.1±328.28	804.51±412.73	1332.65±176.26	<0.01
N-Acryloylmorpholine	5117-12-4	223.85±76.52	447.78±144.86	288.27±181.61	337.59±47.28	0.026
Dimethyl trisulfide	3658-80-8	48788.02±40168.66	968173.1±25504.4	530102.75±411591.69	36590.3±23960.76	0.002

Compound name	CAS	AP	APHP	HP	HPAP	*P*-value
Dimethyl disulfide	624-92-0	58302.10±8451.76	341561.5±21130.18	442956.67±27380.40	293490.87±87267.21	<0.01
Dimethyl sulfone	67-71-0	5071.14±855.73	2123.96±553.62	6770.37±5383.65	2069.2±316.37	0.174
Acetamide	60-35-5	3216.02±350.18	1816.29±207.19	2559.78±135.79	1766.77±104.53	<0.01
Phenanthrene	85-01-8	668.81±82.83	413.77±7.12	499.19±54.36	355.60±4.48	<0.01
Safrole	94-59-7	44.42±16.89	25.54±6.70	60.34±62.38	402.45±298.70	0.047
Dimethylsulfoxide	67-68-5	32.1±24.11	22.02±3.35	26.57±13.00	26.62±6.65	0.857
Diisobutyl fiber solvent		163842.43±29443.87	55582.22±7364	86888.79±13639.19	31707.65±5275.24	<0.01
Fennel Brain	104-46-1	320.32±49.99	93.86±11.73	305.74±45.74	333.16±38.15	<0.01
4,5-Dimethyloxazole	20662-83-3	1196.55±1766.65	501.59±387.66	111.08±82.67	27.46±7.89	0.425
1,2-Dimethylhydrazine	540-73-8	55006.07±1203.91	69272.8±23362.23	53378.38±17030.72	46449.85±13328.71	0.409
methylsulfonyl chloride	124-63-0	206.76±203.27	929.48±287.90	50936.27±86636.97	2905.93±267.67	0.447
infra-methyl sulfide complexes	13292-87-0	17063.99±995.72	13.01±7.51	87.8±85.71	52.53±7.34	<0.01
Eucalyptus Brain	470-82-6	269758.89±269033.21	190781.02±174086.78	148347.65±128786.70	169942.74±159879.67	0.871
		634222.77	1633823.85	1326671.69	587821.42	

Note: HP refers to high-pressure cooking, APHP refers to atmospheric pressure high-pressure cooking, HPAP refers to high-pressure atmospheric pressure cooking. CAS refers to chemical abstracts service.

Table S7. Volatile ingredients of Tibetan sheep (GC-MS, VIP> 1.5, *P* <0.05)

Compound	Quantity contained（mg/kg）				*P*-value	VIP value	Oder characteristic
	AP	APHP	HP	HPAP			
Dimethyl trisulfide	48788.02±40168.66	968173.1±25504.4	530102.75±411591.69	36590.3±23960.76	0.002	5.03824	Sulfur, fish, cabbage

Ethanone, 1-(1H-pyrrol-2-yl)-	11072.69±5132.94	203862.4±21077.4	1183251.67±97282.39	645692.57±237120.26	<0.01	4.89817	Nuts, walnuts, bread
1-Butanol, 3-methyl-	709832.63±25183.31	375647.27±12127.6	105855.32±89314.28	329708.3±16604.6	<0.01	3.18343	Whisky, malt, burnt flavor
Isopropyl Alcohol	226500.43±57321.34	12838.68±4079.21	134150.52±110912.08	418492.3±58285.9	0.001	3.0408	Mushroom flavor.
1-Octen-3-ol	319718.37±65273.15	109488±6179.3	298325.77±41738.32	36844.95±11452.35	<0.01	2.9883	Mushroom flavor
Indole	17156.53±13214.43	24417.96±680.43	349518.67±128830.06	303492.23±86653.8	0.001	2.85284	Mothballs, burnt
Limonene	330037.35±81603.55	64.99±24.59	3482.98±3290.36	104.69±46.15	<0.01	2.70385	Lemon flavor
Bicyclo[2.2.1]heptane, 7,7-dimethyl-2-methylene-	35199.47±29342.38	343726.43±38251.19	446588±46678.7	314887.8±70143.9	<0.01	2.64551	-
Disulfide, dimethyl	58302.1±8451.76	341561.5±21130.18	442956.67±27380.4	293490.87±87267.21	<0.01	2.59894	Onion, cabbage, rancid
Butanoic acid, 3-methyl-, ethyl ester	47.4±0.19	65703.33±21328.26	2327.24±1017.01	167666.17±39067.2	<0.01	2.18419	Fruity
3-Octanone	131102.14±96714.86	168745.9±8290.79	274564.5±23213	75262.3±38866.45	0.011	2.10935	Soap, gasoline
beta-Terpinene	113651.59±37011.52	14414.6±11983.76	154459.57±6390.44	20614.47±28373.47	0	2.10047	Gasoline, turpentine.
Diazene, dimethyl-	149448.4±118563.03	8631.83±7780.05	187326.03±22739.76	126898.07±16487.7	0.034	1.99586	Wine, onion flavor.
1-Butanol, 2-methyl-	24.99±13.43	27058.77±12182.24	160391±26621.07	33532.74±3544.51	<0.01	1.82757	Wine, onion flavor
Diisobutyl cellosolve	163842.43±29443.87	55582.22±7364	86888.79±13639.19	31707.65±5275.24	<0.01	1.72801	Wine, onion flavor.
Bicyclo[4.2.0]octa-1,3,5-triene	801.36±800.94	102699.39±15138	174212.37±51042.73	70015.51±26609.7	0.001	1.71666	-
3-Octanol	13562.46±5238.27	92612.89±37726.41	180368.1±72052.34	139154.97±33521.51	0.009	1.70135	Nuts, mushrooms
6-Nonenal, (Z)-	99898.19±21732.49	33381.57±3437.91	94935.53±13920.62	11649.48±3949.83	<0.01	1.68476	-
Cyclohexanol, 1-methyl-4-(1-methylethenyl)-, acetate	149580.9±36067.3	99865.94±94424.36	69941.31±18072.51	1031.15±485.91	0.042	1.59072	Nuts, mushrooms--

Allyl acetate	112491.99±37104.88	62836.56±6669.45	53.85±48.46	10.8±14.54	<0.01	1.50245	--

Note:AP refers to atmospheric pressure cooking, HP refers to high-pressure cooking, APHP refers to atmospheric pressure high-pressure cooking, HPAP refers to high-pressure atmospheric pressure cooking;VIP refers to variable influence on projection
